# *Flavobacterium flabelliforme* sp. nov. and *Flavobacterium geliluteum* sp. nov., Two Multidrug-Resistant Psychrotrophic Species Isolated From Antarctica

**DOI:** 10.3389/fmicb.2021.729977

**Published:** 2021-10-22

**Authors:** Stanislava Králová, Hans-Jürgen Busse, Matěj Bezdíček, Megan Sandoval-Powers, Markéta Nykrýnová, Eva Staňková, Daniel Krsek, Ivo Sedláček

**Affiliations:** ^1^Department of Experimental Biology, Czech Collection of Microorganisms, Faculty of Science, Masaryk University, Brno, Czechia; ^2^Institut für Mikrobiologie, Veterinärmedizinische Universität Wien, Vienna, Austria; ^3^Department of Internal Medicine – Hematology and Oncology, University Hospital Brno, Brno, Czechia; ^4^Department of Internal Medicine – Hematology and Oncology, Masaryk University, Brno, Czechia; ^5^Department of Biological Sciences, Auburn University, Auburn, AL, United States; ^6^Department of Biomedical Engineering, Faculty of Electrical Engineering and Communication, Brno University of Technology, Brno, Czechia; ^7^NRL for Diagnostic Electron Microscopy of Infectious Agents, National Institute of Public Health, Prague, Czechia

**Keywords:** Antarctica, psychrotrophic bacteria, cold-adaptation, phylogenomics, systematics, *Flavobacterium flabelliforme* sp. nov., *Flavobacterium geliluteum* sp. nov.

## Abstract

Despite unfavorable Antarctic conditions, such as cold temperatures, freeze-thaw cycles, high ultraviolet radiation, dryness and lack of nutrients, microorganisms were able to adapt and surprisingly thrive in this environment. In this study, eight cold-adapted *Flavobacterium* strains isolated from a remote Antarctic island, James Ross Island, were studied using a polyphasic taxonomic approach to determine their taxonomic position. Phylogenetic analyses based on the 16S rRNA gene and 92 core genes clearly showed that these strains formed two distinct phylogenetic clusters comprising three and five strains, with average nucleotide identities significantly below 90% between both proposed species as well as between their closest phylogenetic relatives. Phenotyping revealed a unique pattern of biochemical and physiological characteristics enabling differentiation from the closest phylogenetically related *Flavobacterium* spp. Chemotaxonomic analyses showed that type strains P4023^T^ and P7388^T^ were characterized by the major polyamine *sym-*homospermidine and a quinone system containing predominantly menaquinone MK-6. In the polar lipid profile phosphatidylethanolamine, an ornithine lipid and two unidentified lipids lacking a functional group were detected as major lipids. These characteristics along with fatty acid profiles confirmed that these species belong to the genus *Flavobacterium*. Thorough genomic analysis revealed the presence of numerous cold-inducible or cold-adaptation associated genes, such as cold-shock proteins, proteorhodopsin, carotenoid biosynthetic genes or oxidative-stress response genes. Genomes of type strains surprisingly harbored multiple prophages, with many of them predicted to be active. Genome-mining identified biosynthetic gene clusters in type strain genomes with a majority not matching any known clusters which supports further exploratory research possibilities involving these psychrotrophic bacteria. Antibiotic susceptibility testing revealed a pattern of multidrug-resistant phenotypes that were correlated with *in silico* antibiotic resistance prediction. Interestingly, while typical resistance finder tools failed to detect genes responsible for antibiotic resistance, genomic prediction confirmed a multidrug-resistant profile and suggested even broader resistance than tested. Results of this study confirmed and thoroughly characterized two novel psychrotrophic *Flavobacterium* species, for which the names *Flavobacterium flabelliforme* sp. nov. and *Flavobacterium geliluteum* sp. nov. are proposed.

## Introduction

For a long time, Antarctica was considered an inhospitable environment with low biodiversity. Although it is a part of Earth’s cryosphere which covers about 20% of the Earth’s surface (Boetius et al., 2015), it has been of marginal scientific interest due to its difficult accessibility. Until recently, the majority of information on cold-adapted microorganisms was driven from studies originated in Alpine and Arctic areas. Fortunately, advances in technology have paved the way for scientific research in Antarctica which became an attractive place to conduct polar research. As a result, our knowledge on the Antarctic biodiversity predominated by microorganisms has exponentially raised over the recent decades.

Microbial diversity in Antarctica is surprisingly high despite harsh, extreme conditions that living organisms must endure. Many psychrophilic and psychrotrophic taxa have been successfully isolated from Antarctic environments with high abundance of members of the phylum *Bacteroidetes* (Aislabie et al., 2008; Li et al., 2019). The genus *Flavobacterium* is one of the most frequently isolated genera from this phylum, with an extensive number of cold-adapted species. The genus *Flavobacterium* has been constantly growing over the past years and currently comprises 250 established species (LPSN, accessed 27.05.2021) (Parte et al., 2020). Its members are widely distributed, colonizing significantly diverse niches (stream water, lakes, glaciers, soils, rhizosphere or plants) with an obvious preference to cold environments, including Arctic and Antarctic areas (Bernardet and Bowman, 2015). In addition to their ecological importance, some members of the genus *Flavobacterium* are pathogenic to fish and responsible for considerable economic losses (Wahli and Madsen, 2018).

Wide distribution of *Flavobacterium* spp. in Antarctica is supported by their adaptation and coping mechanisms required for survival in harsh conditions including low temperatures, lack of nutrients or intense UV exposure, and freeze-thaw cycles that exert a strong evolutionary pressure on microbial cells (Kumar et al., 2013). Understanding the strategy these microbes employ to survive in cold environments is now increasingly possible due to genomic comparisons of cold-adapted bacteria and their mesophilic relatives. Genes related to the adaptation in psychrophilic and psychrotrophic *Flavobacterium* spp. encode proteorhodopsins, ice-binding proteins, extracellular polysaccharides, and proteins removing reactive oxygen species or cold-shock proteins (Liu et al., 2019). Products of these genes were found useful in biotechnological applications, food-industry, or bioremediation (Cavicchioli et al., 2011) which proportionally increases research interests in cold-adapted microorganisms. The fact that Antarctica represents an extreme and understudied habitat opens many possibilities to explore not only microbial diversity, but also microbial cold-adaptation mechanisms and potential biotechnological applications of its native microbiota. Taxonomic, ecological, physiological and biotechnology-related questions make unique Antarctic *Flavobacterium* spp. an attractive object to study. In addition, the unique resistome of flavobacterial isolates cultivated from a pristine Antarctic environment makes these microorganisms an important model for studying antibiotic resistance beyond the clinical context (González-Aravena et al., 2016; Jara et al., 2020). As such, Antarctic species with novel mechanisms of resistance, including the two novel *Flavobacterium* species described in this study, represent an important source of antibiotic resistance genes (González-Aravena et al., 2016).

## Materials and Methods

### Isolation, Preservation, Culture Conditions

The present study describes a taxonomic investigation of eight *Flavobacterium* strains (Supplementary Table 1) isolated in the frame of the microbiological research of the Czech Antarctic Research Program (CARP^1^). Microbiological sampling is conducted yearly on the James Ross Island (near the north-eastern extremity of the Antarctic Peninsula), Antarctica. Sampling is performed during the summer period when the majority of the island becomes an ice-free area. Antarctic summer season allows sampling of upper soil layers, permafrost, lakes and proglacial streams, which are all areas of intense microbial activities (Kopalová et al., 2013; Nedbalová et al., 2013). *Flavobacterium* spp. analyzed in this study were isolated from environmental materials (different water, organic and soil sources) sampled during the years 2010–2019 (Supplementary Table 1). Water samples were spread on Reasoner’s 2A agar plates (R2A, Oxoid) (150 μl) and cultivated at 15°C for 5 days. Soil samples were processed by dispersing 1 g of soil and/or stone fragment material in 5 ml of sterile saline solution followed by dispersing of 100 μl of obtained solution on R2A agar plates and cultivated at 15°C for 7 days. Large numbers of well-separated yellow pigmented colonies were randomly selected, hand-picked by sterile inoculation sticks and purified by subculturing. Pure cultures were maintained on the R2A agar slants until transportation to the Czechia. After transportation, subcultures were checked for purity and maintained at −70°C for long-term storage. The type strains of the phylogenetically closest relatives *Flavobacterium hercynium* CCM 9054^T^, *Flavobacterium branchiicola* CCM 9061^T^, *Flavobacterium chilense* CCM 7940^T^, *Flavobacterium araucananum* CCM 7939^T^, *Flavobacterium psychroterrae* CCM 8827^T^ and *Flavobacterium saccharophilum* CCM 8770^T^ were obtained from the Czech Collection of Microorganisms (CCM^2^) for parallel testing and comparison purposes. All strains were routinely cultivated on R2A agar plates at 20°C for 48 hrs.

### Phylogenetic Analyses

Genomic DNA was extracted and purified using High Pure PCR Template Preparation Kit (Roche Diagnostics) according to the manufacturer’s recommendations. The 16S rRNA genes were amplified and sequenced using universal bacterial primers pA (5′-AGAGTTTGATCCTGGCTCAG-3′) and pH (5′-AAGGAGGTGATCCAGCCGCA-3′) (Edwards et al., 1989). PCR products were purified using High Pure PCR Product Purification Kit (Roche Diagnostics). Sequencing was performed by the Eurofins MWG Operon sequencing facility^3^. The 16S rRNA sequences were submitted to the EzBioCloud server (Yoon et al., 2017) for initial identification and determination of the closest phylogenetic neighbors. The 16S rRNA sequences of the phylogenetically closest and validly named species were downloaded from the GenBank database^4^ and used for multiple sequence alignment using the Molecular Evolutionary Genetics Analysis (MEGA) software (v7.0) (Tamura et al., 2013). The maximum-likelihood (ML) method with the Tamura-Nei gamma distance model was used to calculate the genetic distances and to build the phylogenetic tree.

### Whole Genome Sequencing

Genomic DNA was extracted using a High Pure PCR Template Preparation Kit as described above. Sequencing libraries were prepared using KAPA HyperPlus Kits (Roche, Switzerland) with 7 min of enzymatic fragmentation and final fragments length within the range of 550–650 bp. Sequencing was performed on Illumina MiSeq platform with MiSeq Reagent Kit v2 (500-cycles) (Illumina, United States). The St. Petersburg genome assembler (SPAdes v3.11.1) (Bankevich et al., 2012) was used for *de novo* genome assembly. The MismatchCorrector was run to reduce short indels and the number of mismatches. The read coverage cut-off value was automatically computed by the software resulting in contigs and scaffolds. Quality assessment was performed by the QUAST tool (Gurevich et al., 2013) and Bowtie2 v2.4.2 (Langmead and Salzberg, 2012).

### Phylogenomics and Genomics

The whole genome sequence (WGS) data were analyzed for further confirmation of the taxonomic status of the analyzed strains and for comparison with genomes of related *Flavobacterium* spp. The WGS data of type strains P4023^T^ and P7388^T^ were submitted to the Type Strain Genome server (TYGS) (Meier-Kolthoff and Göker, 2019) and to the Microbial Genome Atlas (MiGA) (Rodriguez-R et al., 2018). To further confirm the taxonomic status of the analyzed strains, type strain genomes along with genomes of the closest related and validly described *Flavobacterium* spp. were subjected to the UBCG pipeline (Na et al., 2018) with default parameters to calculate a phylogenetic tree based on 92 concatenated core genomes. The whole genome sequences of the closest *Flavobacterium* spp. were downloaded from the GenBank database and the genomes of *F. branchiicola* CCM 9061^T^ and *F. psychroterrae* CCM 8827^T^ were sequenced and assembled in this study as described above and deposited in the GenBank database under accession numbers GCA_018383905.1 and GCA_018380615.1, respectively. Average nucleotide identity (ANI) values were calculated using the ANI calculator on the Kostas Laboratory website^5^ using reciprocal best hits (two-way ANI) (Rodriguez-R and Konstantinidis, 2016). Protein-coding sequences were calculated using the GeneMarkS prediction tool (Besemer et al., 2001) and subjected to the amino acid average identity values (AAI) calculator on the Kostas Laboratory website^6^ (Rodriguez-R and Konstantinidis, 2016). Digital DNA-DNA hybridization (dDDH) values were generated using the Genome to Genome Distance Calculator (GGDC) version 2.1 (Meier-Kolthoff et al., 2013).

Additional genome annotation was performed using the Prokaryotic Genome Annotation Pipeline (PGAP) (Tatusova et al., 2016; Li et al., 2021) and Operon-Mapper (Taboada et al., 2018). Functional annotation was performed with EggNOG-mapper v5.0 (Huerta-Cepas et al., 2019). CRISPRs were identified by CRISPR Detect (Biswas et al., 2016). Presence of putative prophages was predicted by PHASTER (Arndt et al., 2016) and Prophage Hunter (Song et al., 2019) tools. Genome mining for potential secondary-metabolite associated biosynthetic gene clusters (BGCs) was determined using antiSMASH version 6.0.0 (Blin et al., 2021). Annotations of predicted open reading frames (ORFs) and genes/domains from candidate BGCs were visualized using the R package “gggenes” (Wilkins and Kurtz, 2019) and “ggplot2” (Wickham, 2016). ABRicate v1.0.1^7^ was used for screening of known antibiotic and metal resistance genes using the ResFinder database (Zankari et al., 2012), NCBI Bacterial Antimicrobial Resistance Reference Gene Database, CARD (Jia et al., 2017), MEGARes database (Doster et al., 2020) and ARG-ANNOT tool (Gupta et al., 2014). Further *in silico* prediction of resistomes and putative resistance genes was performed using Resistance Gene Identifier (RGI, v5.1.1)^8^ with CARD v3.1.1 database (Jia et al., 2017). The enzymatic potential for carbohydrate degradation was evaluated by the web-based tools dbCAN2 and dbCAN-PUL for automated carbohydrate-active enzyme annotation (Yin et al., 2012; Zhang et al., 2018; Ausland et al., 2021).

### Morphology

The colony morphology was determined on R2A agar after 48 h cultivation at 20°C. The cellular morphology of all strains was observed by light microscopy after Gram staining. The cellular morphology of the type strains, P4023^T^ and P7388^T^, was also examined by transmission electron microscopy using a Morgagni 268D Philips (FEI Company) electron microscope.

### Temperature, NaCl and pH Tolerance

The growth at different temperatures (1–40°C, in 5°C increments) and the salt tolerance (0.5, 1.0, 2.0, 3.0, 4.0 of NaCl) were determined on R2A agar plates. The pH tolerance was assessed in Trypticase Soya Broth (TSB) inoculated with two drops of cell suspension of concentration as McFarland 2.0 with pH 5–10 at intervals of 1.0 pH unit at 20°C adjusted with the following buffer systems: pH 5.0–8.0, 0.1 M KH_2_PO_4_/0.1 M NaOH; pH 9.0–10.0, 0.1 M NaHCO_3_/0.1 M Na_2_CO_3_. The pH value of the TSB was confirmed after autoclaving.

### Biochemical and Physiological Characteristics

All eight isolates along with reference strains *F. hercynium* CCM 9054^T^, *F. branchiicola* CCM 9061^T^, *F. chilense* CCM 7940^T^, *F. araucananum* CCM 7939^T^, *F. psychroterrae* CCM 8827^T^ and *F. saccharophilum* CCM 8770^T^ were phenotypically characterized by the most relevant tests recommended for description of new taxa within the family *Flavobacteriaceae* (Bernardet et al., 2002). These tests comprised of: activities of oxidase (OXItest; Erba-Lachema) and catalase (Atlas, 2010) (ID color Catalase; bioMérieux) performed according to the manufacturer’s instructions; production of urease (Christensen, 1946); production of arginine dihydrolase, ornithine and lysine decarboxylase production (Brooks and Sodeman, 1974), oxidation-fermentation test (Hugh and Leifson, 1953); production of acid from carbohydrates aerobically (Gilardi, 1985), production of H_2_S on Triple Sugar Iron Agar (HiMedia), hydrolysis of aesculin, starch (Barrow and Feltham, 1993), gelatine, Tween 80 (Pacova and Kocur, 1984), casein, tyrosine (Kurup and Babcock, 1979), cellulose (R2A broth with strip of Whatman paper No.1) (Ali et al., 2009), carboxymethylcellulose (CMC) (Ibrahim et al., 2021) and O-nitrophenyl-β-D-galactopyranoside (ONPG) (Lowe, 1962), phospholipinase activity in the egg-yolk reaction (Owens, 1974), nitrate and nitrite reduction, production of indole, utilization of citrate in Simmon’s citrate agar (Barrow and Feltham, 1993), utilization of acetamide (Oberhofer and Rowen, 1974) and sodium malonate (Ewing, 1960). The presence of flexirubin-type pigments was tested using 20% (w/v) KOH solution and the presence of capsule was identified by the Congo red adsorption test (Bernardet et al., 2002).

Capability of growth on different media was tested on R2A agar (Oxoid), Nutrient agar (Oxoid), Plate Count agar (Oxoid), Tryptone Soya agar (Oxoid), marine agar (Lapage et al., 1970), MacConkey and Endo agar (HiMedia), Brain Heart Infusion agar (Oxoid), and Mueller-Hinton agar (Oxoid) under aerobic conditions. Anaerobic growth was tested using Anaerocult A system (Merck) and microaerophilic growth in atmosphere of 5% CO_2_, both on R2A agar plates. All above listed tests were inoculated using cells of analyzed strains grown on R2A agar at 20°C for 48 hrs. All tests were read daily for up to 7 days with exception of the tyrosine hydrolysis test (up to 10 days). The utilization of carbon sources, enzyme activities and other additional phenotypic characteristics were further tested using identification test kits GEN III MicroPlateTN (Biolog) with the protocol A, API 20 NE and API ZYM (bioMérieux) according to the manufacturer’s instructions.

### Antimicrobial Susceptibility

*In vitro* antimicrobial susceptibility was assessed using the disk diffusion method. Cell suspensions of concentration as 0.5 McFarland were prepared from cultures cultivated on R2A agar. Suspension containing each strain (100 μl) was spread on the Mueller-Hinton agar plates. Tested antibiotic disks (Antimicrobial Susceptibility Disks, Oxoid) were as follows: ampicillin (10 μg), aztreonam (30 μg), carbenicillin (100 μg), cefixime (5 μg), ceftazidime (10 μg), cephalotin (30 μg), ciprofloxacin (5 μg), gentamicin (10 μg), chloramphenicol (30 μg), imipenem (10 μg), kanamycin (30 μg), co-trimoxazole (25 μg), piperacillin (30 μg), polymyxin B (300 U), streptomycin (10 μg) and tetracycline (30 μg). CLSI (The Clinical and Laboratory Standards Institute) and EUCAST (The European Committee on Antimicrobial Susceptibility Testing) standards were strictly followed for cultivation and inhibition zone diameter reading (CLSI, 2015; EUCAST, 2017).

### Chemotaxonomic Analyses

Cellular fatty acid methyl esters analysis was performed from biomass of all strains including the reference type strains incubated strictly under the same conditions. All cultures were grown on R2A agar at 20 ± 2°C for 48 hrs to reach the late-exponential stage of growth according to the four quadrant streak method (Sasser, 1990). Fatty acids were identified using an Agilent 7890B gas chromatograph according to the Standard Protocol of the Sherlock Identification System (MIDI Sherlock version 6.2, MIDI database RTSBA 6.21). Further chemotaxonomic analyses including polar lipids, quinones, and polyamines were performed using freeze-dried biomass prepared from cells grown in R2A broth at 20°C for 48 hrs. Extraction of all above mentioned chemotaxonomic markers was performed according to the previously described protocols (Busse and Auling, 1988; Tindall, 1990b, a; Altenburger et al., 1996; Stolz et al., 2007). Analysis of the polyamine patterns was performed using high-performance liquid chromatography (HPLC) with conditions described by Busse et al. (1997) using the HPLC apparatus as described previously (Stolz et al., 2007).

## Results and Discussion

### Phylogenetic Analysis

Initial identification based on the 16S rRNA gene sequences classified all Antarctic isolates to the genus *Flavobacterium*. Based on the 16S rRNA gene sequence similarity, Antarctic isolates formed two groups for which strains P4023^T^ and P7388^T^ were assigned as type strains. The closest related species to proposed type strain P4023^T^ were *F. saccharophilum* DSM 1811^T^ (98.54%), *F. hercynium* DSM 18292^T^ (98.54%) and *F. chilense* LMG 26360^T^ (98.39%). A proposed type strain for the second cluster, P7388^T^, showed the highest sequence similarities to the *F. branchiicola* 59B-3-09^T^ (99.12%), *F. araucananum* DSM 24704^T^ (99.08%) and *F. psychroterrae* CCM 8827^T^ (99.04%). The 16S rRNA gene sequence similarities of strain P4023^T^ fell below the cut-off value 98.65%, whereas strain P7388^T^ reached similarities above this threshold. Nevertheless, several studies have already proved that the standard 16S rRNA gene sequence cut-off value is not sufficient for some genera, such as *Streptomyces, Streptococcus* or *Bacillus* (Fox et al., 1992; Clarridge, 2004; Guo et al., 2008). Indeed, the genus *Flavobacterium* and its species with validly published names also do not follow the 98.65% threshold, which therefore requires additional in-depth phylogenetic and phylogenomic analyses for designation of *Flavobacterium* spp. taxonomic positions and assessment of their novelty (Yi and Chun, 2006; Romanenko et al., 2015).

**FIGURE 1:**
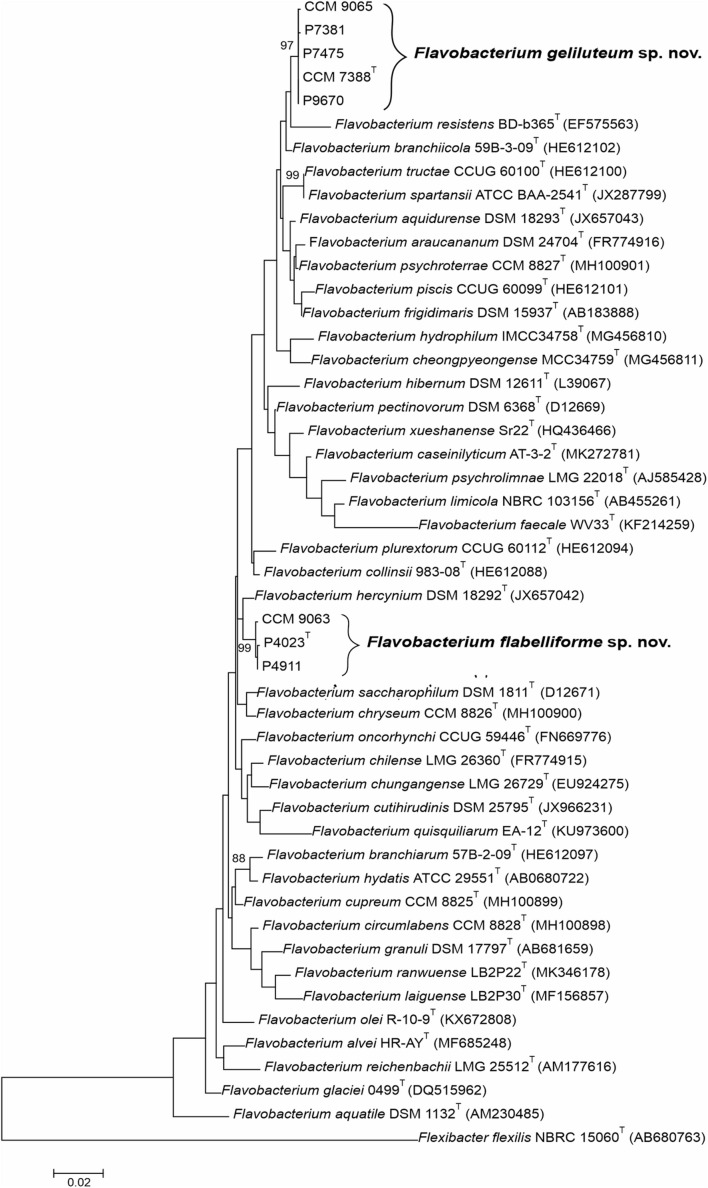
Phylogenetic tree based on 16S rRNA gene sequences comparison showing the phylogenetic positions of *Flavobacterium flabelliforme* sp. nov. and *Flavobacterium geliluteum* sp. nov. among their closest related species within the genus *Flavobacterium*. The evolutionary history was inferred by using the maximum likelihood method based on Tamura-Nei distance with the gamma model. All positions with less than 95% site coverage were eliminated. Bootstrap probability values (percentages of 1,000 tree replications) greater than 70% are indicated at branch points. *Flexibacter flexilis* NBRC 15060^T^ (AB680763) was used as an outgroup. Bar, 0.02 substitutions per nucleotide position.

To evaluate phylogenetic relationships between Antarctic *Flavobacterium* spp. and their closest phylogenetic neighbors as identified using the EzBioCloud database, the ML phylogenetic tree was calculated and clearly showed well-supported clades comprising of strains P4023^T^ and P7388^T^ along with two and four non-type isolates, respectively (Figure 1). Strains P4023^T^, P4911 and CCM 9063 formed a separate cluster with the closest phylogenetic neighbor *F. hercynium* DSM 18292^T^ supporting the 16S rRNA gene sequence similarities results. Strains P7388^T^, P9670, P7381, P7475, and CCM 9065 formed a well-supported cluster close to *Flavobacterium resistens* BD-b365^T^ and *F. branchiicola* 59B-3-09^T^.

### Phylogenomics

**FIGURE 2:**
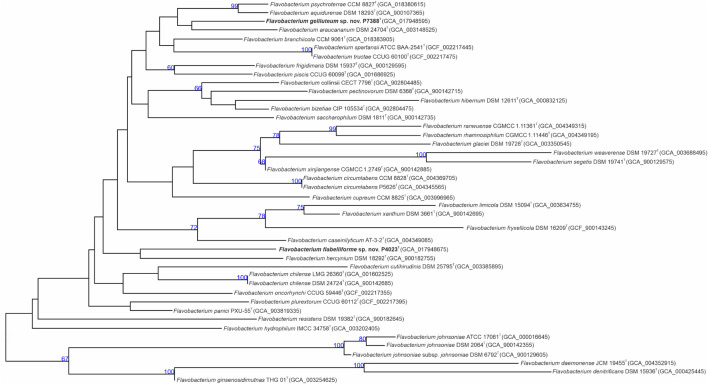
Phylogenomic tree inferred with FastME 2.1.6.1 (Lefort et al., 2015) from GBDP distances calculated from genome sequences. Branch lengths are scaled in terms of GBDP distance formula d_5_. Numbers above branches indicate GBDP pseudo-bootstrap support values from 100 replications with the average branch support of 85.1%.

As mentioned above, clear taxonomic delineation of *Flavobacterium* spp. requires additional comprehensive analyses. For this reason, draft genomes of proposed type strains P4023^T^ and P7388^T^ were initially submitted to TYGS and MiGA platforms for genomic comparisons. TYGS results assigned both strains as novel species. Genome-based phylogeny calculated by TYGS showed *F. hercynium* DSM 18292^T^ as the closest phylogenetic neighbor of strain P4023^T^, and *F. araucananum* DSM 27404^T^ forming a monophyletic cluster with strain P7388^T^ (Figure 2). MiGA results confirmed novelty of both draft genomes and assigned *Flavobacterium weaverense* DSM 19727^T^ and *Flavobacterium pectinovorum* ATCC 19366^T^ as the closest related species to P4023^T^ and P7388^T^, respectively. Further phylogenomic analysis through the up-to-date bacterial core gene UBCG pipeline placed strain P4023^T^ into a cluster shared with *F. weaverense* DSM 19727^T^, while strain P7388^T^ formed a distinct phylogenetic lineage within the genus *Flavobacterium* (Supplementary Figure 1).

*In silico* comparison of genomic distances and calculation of the dDDH values between the draft genomes of strains P4023^T^, P7388^T^ and their closest phylogenetic neighbors resulted in values below 70%, the cut-off established as a threshold value for species delineation by dDDH (Meier-Kolthoff et al., 2013). The ANI values between strain P4023^T^ and its closest relatives reached 77.60–82.70% with the highest nucleotide similarity to *F. weaverense* DSM 19727^T^. Comparison between strain P7388^T^ and its closest phylogenetic relatives showed ANI values of 78.17–82.06% with *F. araucananum* DSM 24704^T^ with the highest genomic relatedness. Calculated ANI values were well below the 95–96% threshold for species delineation and further supported novelty of the proposed species (Ciufo et al., 2018; Jain et al., 2018). This was also consistent with the calculated AAI values ranging from 71.24–84.84% and 72.77–82.09% for strains P4023^T^ and P7388^T^, respectively (Table 1).

**TABLE 1:** ANI, AAI, and dDDH genomic comparisons between strains P4023^T^, P7388^T^ and their closest phylogenetic neighbors.

Reference genomes	GenBank accession number	ANI value (%)		AAI value (%)		dDDH	
		P4023^T^	P7388^T^	P4023^T^	P7388^T^	P4023^T^	P7388^T^
*F. hercynium* DSM 18292^T^	GCA_002217285.1	79.29	81.30	71.87	80.46	20.8	23.3
*F. chilense* DSM 24724^T^	GCA_900142685.1	78.84	81.48	71.24	81.10	20.4	23.4
*F. saccharophilum* DSM 1811^T^	GCA_900142735.1	79.40	82.02	72.32	82.09	21.1	23.9
*F. branchiicola* CCM 9061^T^	GCA_018383905.1	79.22	81.35	71.24	80.05	21.0	24.4
*F. araucananum* DSM 24704^T^	GCA_003148525.1	79.15	82.06	72.13	81.88	21.0	24.4
*F. psychroterrae* CCM 8827^T^	GCA_018380615.1	79.50	82.01	72.09	81.25	21.4	22.9
*F. weaverense* DSM 19727^T^	GCA_003688495.1	82.70	78.72	84.84	72.77	24.6	20.4
*F. pectinovorum* DSM 6368^T^	GCA_900142715.1	79.45	82.03	71.98	81.74	20.6	24.5
*F. aquatile* LMG 4008^T^	GCA_000757385.1	77.60	78.17	68.34	67.49	19.6	19.6
*F. geliluteum* sp. nov. P7388^T^	GCA_017948595.1	79.44	–	78.89	–	20.6	–
*F. flabelliforme* sp. nov. P4023^T^	GCA_017948675.1	–	79.44	–	78.89	–	20.6

### Genomic Analyses

#### General and Functional Features of P4023^T^ Genome

The final genome assembly of strain P4023^T^ contained 57 scaffolds with a draft genome size of 3,631,245 bp and the genomic G + C content 31.2 mol% (Supplementary Table 2). PGAP predicted 3,178 genes in total, among which 3,090 belonged to protein-coding genes (CDSs) with 2,525 (81.72%) assigned to a function with high confidence and 565 (18.28%) assigned as hypothetical proteins. The genome contained 48 tRNAs, seven rRNAs (three 5S rRNAs, three 16S rRNAs, one 23S rRNA) and three ncRNAs. The genome does not contain plasmids or CRISPR arrays. Interestingly, seven prophages with four assigned as active were detected by Prophage Hunter and one additional prophage was found by the PHASTER tool.

Functional annotation of the genomes revealed that 359 genes (11.60%) could not be assigned to a specific class of orthologous genes clusters (COGs) or with specific function (667 genes, 21.55%) (Supplementary Table 3). However, the remaining majority of genes (67.25%) were assigned to specific COGs classes with the most abundant classes as (M) Cell wall/membrane/envelope biogenesis, (E) Amino acid transport and metabolism, (J) Translation, ribosomal structure and biogenesis, (K) Transcription and (L) Replication, recombination and repair, followed by (C) Energy production and conversion, (P) Inorganic ion transport and metabolism, and (H) Coenzyme transport and metabolism. In total, 76 carbohydrate-active enzymes (CAZymes) were predicted comprising 17 families of glycoside hydrolases (GHs), four families of carbohydrate esterases (CEs), seven families of glycosyltransferases (GTs), and single carbohydrate-binding modules (CBMs) family. Two polysaccharide utilization loci (PULs), PUL0344 and PUL0487, were further predicted, both associated with chitin degradation of *Flavobacterium johnsoniae* (McBride et al., 2009; Larsbrink et al., 2016).

#### General Features of P7388^T^ Genome

The final genome assembly of strain P7388^T^ contained 112 scaffolds with a draft genome size of 4,387,206 bp and the genomic G + C content 34.5 mol% (Supplementary Table 2). PGAP predicted 3,829 genes in total, among which 3,728 belonged to protein-coding genes (CDSs) with 2,928 (78.54%) assigned to a function with high confidence and 800 (21.46%) assigned as hypothetical proteins. The genome contained 46 tRNAs, three rRNAs (one of each 5S rRNA, 16S rRNA and 23S rRNA) and three ncRNAs. No plasmids were found in the genome. One CRISPR array was identified by CRISPR Detect with medium confidence. A high number of prophages were predicted in the P7388^T^ genome with 14 prophages detected by Prophage Hunter, six of which were predicted to be active. Six additional incomplete prophage sequences were detected by the PHASTER tool.

**FIGURE 3:**
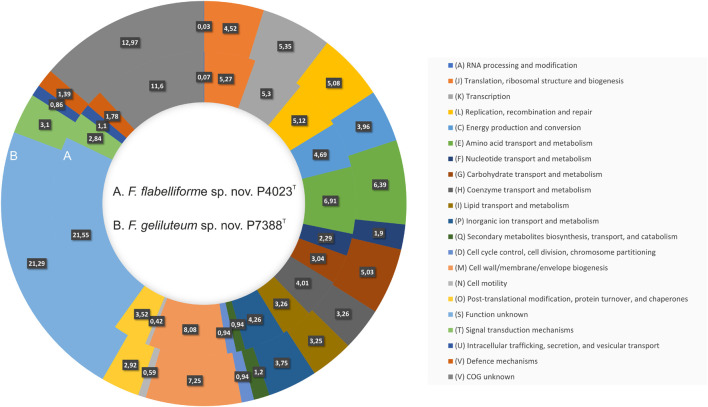
Comparison of COG functional categories between *F. flabelliforme* sp. nov. P4023^T^ and *F. geliluteum* sp. nov. P7388^T^. Each colored segment indicates the relative contribution of a functional category as a percentage of total COGs with the color of COG family indicated in the legend. Ring A, *F. flabelliforme* sp. nov. P4023^T^; Ring B, *F. geliluteum* sp. nov. P7388^T^.

Functional annotation revealed that 485 genes (12.97%) could not be assigned to any COGs or with specific function (796 genes, 21.29%) (Supplementary Table 3). The remaining majority of genes (74.74%) were assigned to specific COGs classes with the most abundant classes annotated as (M) Cell wall/membrane/envelope biogenesis, (E) Amino acid transport and metabolism, (K) Transcription, (L) Replication, recombination and repair, and (J) Translation, ribosomal structure and biogenesis, followed by (C) Energy production and conversion, (P) Inorganic ion transport and metabolism, and (H) Coenzyme transport and metabolism. Comparison of relative abundances of functional COG classes between strains P4023^T^ and P7388^T^ is depicted in Figure 3. The genome of P7388^T^ was predicted to harbor significantly more CAZymes with 176 in total, compared to 76 from P4023^T^. Overall, P7388^T^ harbors 41 families of GHs, nine families of CEs, 10 families of GTs, seven families of CBMs and two polysaccharide lyases (PLs). Prediction of PULs found seven putative loci involved in degradation of carrageenan, dextran, hemicellulose, chitin, pectin, starch and xylan, which implies the ability of strain P7388^T^ to degrade various polysaccharide substrates.

#### Biosynthetic Potential of the Novel Strains

**FIGURE 4:**
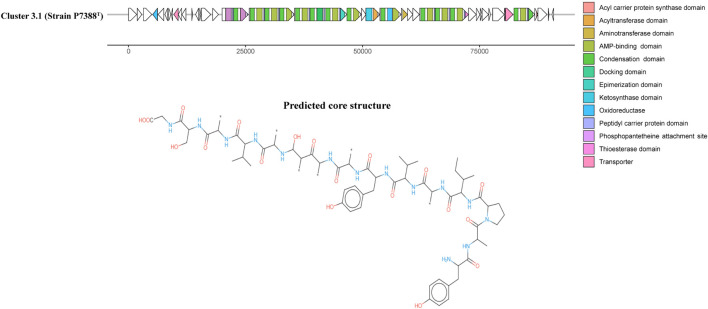
A polyketide synthase (PKS)-non-ribosomal peptide synthetase (NRPS) hybrid BGC, Cluster 3.1, and the predicted core structure annotated by antiSMASH (Blin et al., 2021) from the genome of strain P7388^T^. ORFs are depicted as white arrows and the core PKS-NRPS biosynthetic domains are color coded in each ORF.

**FIGURE 5:**
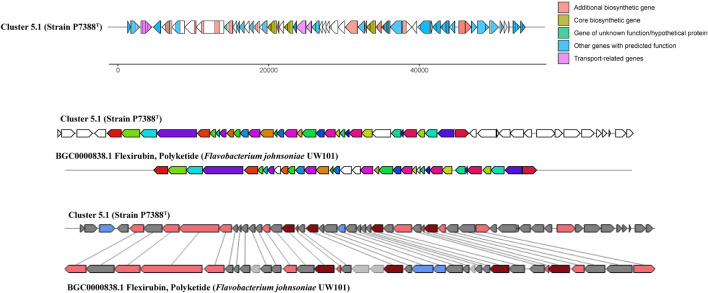
Annotation of an arylpolyene/resorcinol BGC, Cluster 5.1, from strain P7388^T^ based on antiSMASH analysis and similarity of Cluster 5.1 to a PKS flexirubin biosynthetic gene cluster from *Flavobacterium johnsoniae* UW101 (BGC0000838.1).

Antarctic bacteria have been recognized as an understudied source of secondary metabolites (Benaud et al., 2020; Waschulin et al., 2020). AntiSMASH annotation of secondary metabolite biosynthesis was performed to explore the biosynthetic potential of strains P4023^T^ and P7388^T^. The analysis indicated that these strains harbor four (P4023^T^) and seven (P7388^T^) gene clusters potentially related to secondary metabolite production. Out of these, six clusters did not match any known BGCs deposited in the MIBiG database (Kautsar et al., 2020) and other three clusters were below 30% similarity to any known BGCs. Strain P4023^T^ harbors a BGC encoding a terpene with 28% similarity to a carotenoid BGC of *Algoriphagus* sp. KK10202C. Three additional BGCs were categorized as terpene, arylpolyene and betalactone, and none of these clusters showed significant similarity to known BGCs. Arylpolyenes are widely distributed bacterial natural products that are structurally and functionally similar to carotenoid pigments (Schöner et al., 2016), and are particularly important regarding protection from reactive oxidation, a severe threat in Antarctica. No prediction of flexirubin-associated genes suggests that the yellow pigment of P4023^T^ may be of arylpolyene or carotenoid nature. Interestingly, strain P7388^T^ was predicted to harbor more BGCs than strain P4023^T^, and the number of predicted BGCs was similar to *F. chilense* LMG 26360^T^ and *F. hercynium* DSM 18292^T^. Among these BGCs, a core structure was predicted from one hybrid polyketide synthase (PKS)-non-ribosomal peptide synthetase (NRPS) BGC for strain P7388T that did not match any known BGCs (Cluster 3.1, Figure 4). Only one putative BGC from strain P7388^T^, Cluster 5.1, encoding an arylpolyene/resorcinol pathway showed >50% similarity to a known cluster. Annotation of Cluster 5.1 (Figure 5) revealed numerous PFAM hits with genes involved in the core biosynthetic process such as Beta-ketoacyl synthases, as well as transport-related genes, glycosyl transferases, and carbohydrate kinases. Comparison of Cluster 5.1 to the MiBIG database showed that this cluster was 88% similar to a polyketide cluster encoding a flexirubin-type pigment of *F. johnsoniae* UW101 (Figure 5) along with two terpenes sharing 20% and 28% similarity to carotenoid BGCs of *Algoriphagus* sp. KK10202C and *Streptomyces avermitilis*, respectively. Thus, yellow pigmentation of the strain P7388^T^ is likely a result of a pigment mixture of flexirubin-type and carotenoid pigments resulting in the dark yellow to orange pigmentation of colonies. Furthermore, the production of secondary metabolites, especially activities of PKSs and NRPSs, has been associated with bacteria expressing gliding motility (Nett and König, 2007). The Antarctic *Flavobacterium* strains studied not only were predicted to encode some PKS and NRPS clusters but were also found to express different degrees of gliding motility promoted by lower temperatures (<20°C) (Supplementary Figures 2, 3). This phenotypic characteristic is genomically encoded by genes essential or related to gliding motility and type 9 secretion systems (T9SS) present in the genomes of both P4023^T^ and P7388^T^ and is listed in Supplementary Table 4.

#### Genomic Features Associated With Cold-Adaptation

A number of genes related to cold-adaptation have been identified in type strain genomes of the two proposed Antarctic species and were compared to three phylogenetically related mesophilic flavobacteria, *F. hercynium* DSM 18292^T^, *F. saccharophilum* DSM 1811^T^ and *F. pectinovorum* DSM 6368^T^. Genome-mining focused on cold-adaptation confirmed that *Flavobacterium* spp. are well adjusted to environmental stress and harbor a significant number of genes associated with cold-adaptation regardless of their thermotype (Supplementary Table 5).

Cold-shock-inducible proteins are widely distributed among various bacteria and include cold-shock proteins (), ribosome-binding factor A (*rbfA*), transcription termination/antitermination factor (*nusA*), translation initiation factors IF1 and IF2 (*infA, infB*), polynucleotide nucleotidyltransferase (*pnp*), heat-shock cognate proteins (*hsc*), recombinase A (*recA*) and many others (Gualerzi et al., 2003; Hesami et al., 2011; Barria et al., 2013). Comparison of psychrotrophic and mesophilic genomes showed that presence of these genes is highly conserved among *Flavobacterium* spp. and may be partially responsible for their ubiquitous nature. Most of the cold-induced genes were harbored in all analyzed strains. One copy of a conserved *cspB* gene was found in all analyzed genomes and one copy of *cspC* gene was present in P7388^T^, *F. saccharophilum* DSM 1811^T^ and *F. pectinovorum* DSM 6368^T^. The *cspB* gene isolated from an arctic strain of *Polaribacter irgensii* (KOPRI 22228), a member of family *Flavobacteriaceae*, was found to substantially increase tolerance to freezing and thus considered a primary response to ensure freeze-tolerance of its hosts (Jung et al., 2018). Additionally, all strains encode a trigger factor Tgi, which has been suggested as a main cold-related chaperone of an Antarctic strain, *Pseudoalteromonas haloplanktis* TAC125 (Piette et al., 2010). Additional cold-inducible genes, such as putative cold-shock DEAD-box helicase A (*deaD*), chromosomal replication initiation ATPase (*dnaA*), DNA topoisomerases IV (*gyrA*, *gyrB*), transcription antitermination factor (*nusA*), translation factors IF-1, 2, 3 (*infA, infB, infC*) along with other cold-inducible genes were present in all genomes in one copy, regardless of the thermotype (Supplementary Table 5). Surprisingly, housekeeping genes associated with cold-adaptation, such as sigma factor 70 (*rpoD*) and sigma factor 24 (*rpoE*) were present in genomes only in one and two copies, respectively, although psychrotrophic *Flavobacterium* spp. were previously found to harbor multiple copies of these genes (Liu et al., 2019). Ice-binding (IBP) or antifreeze (ATF) proteins inhibiting formation of ice crystals and their recrystallization were not found in either genome, although gene *ffIBP* encoding IBP was already detected in the genome of psychrophilic marine strain, *Flavobacterium frigoris* PS1 (Kim et al., 2019). As ice-nucleating activity was detected in flavobacteria strains found confined in ice cores (Christner et al., 2000; Wilson et al., 2006), it is likely that genes encoding IPBs and ATFs are present, however, these remain unassigned with hypothetical functions.

Cold-adaptation is inseparably associated with oxidative stress response as a result of higher oxygen solubility at lower temperatures leading to increased levels of reactive oxygen species (ROS). Notably, *Flavobacterium* spp. are well prepared to withstand oxidative stress by the production of proteins and enzymes involved in removal of ROS, such as catalases (KatA, KatG, KatE), superoxide-dismutase and peroxidase (SodA, SodC, Bcp), or thioredoxin and peroxiredoxin reductases (TrxA, TrxB, OsmC, OsmC-like proteins). Although all analyzed strains harbored peroxiredoxin genes in multiple copies (*osmC, osmC*-like), strain P4023^T^ harbored multiple copies of superoxide-dismutase (*sodA*) and thioredoxin (*trxA*), which may be explained by its isolation from surface rather than deeper soil layers and therefore exposed to higher oxidative stress and stronger UV radiation. Similarly versatile microorganisms to flavobacteria are pseudomonads that colonize diverse Antarctic areas including soils, fresh or marine waters (Reddy et al., 2004; Kosina et al., 2013; Jang et al., 2020). Considering they share the same environment, it is not surprising that pseudomonads also share certain anti-oxidative mechanisms with flavobacteria, particularly, various catalases, superoxide-dismutases, thioredoxins, or peroxiredoxins (Orellana-Saez et al., 2019). However, some mechanisms seem to be unique to pseudomonads, such as the expression of glutathione-related proteins and glyoxalase by *Pseudomonas* sp. MPC6 or the production of polyhydroxyalkanoates (PHAs) by *Pseudomonas extremaustralis* 14-3^T^ (Ayub et al., 2009; Tribelli et al., 2020). None of these anti-oxidative proteins have been found in the genome analysis of studied Antarctic strains, which suggests that cold-tolerant bacteria have developed several mechanisms to sustain oxidative stress, and these mechanisms can vary across bacterial taxa including those that co-inhabit the same extreme environment.

Genome analysis further revealed homologs to genes involved in carotenoid biosynthesis among all analyzed strains. Carotenoid pigments are associated with cold-adaptation as regulators of membrane fluidity, protectants against UV radiation and oxidative stress (Baraúna et al., 2017). Interestingly, only P4023^T^ harbored a *crtZ* gene required for zeaxanthin biosynthesis (Pasamontes et al., 1997), while the remaining *Flavobacterium* spp. lacked this gene, suggesting that their carotenoid biosynthetic pathway ends with β-carotene. Interestingly, while yellow coloration of strain P4023^T^ colonies seems to be a result of carotenoid pigments, strain P7388^T^ also harbors a biosynthetic gene cluster encoding flexirubin biosynthesis that may also contribute to its coloration. Phenotypic tests for the presence of flexirubin-type pigments correlated with predicted genomic features, resulting in a negative test result for P4023^T^ and positive result for P7388^T^.

Proteorhodopsin (PR) is a membrane light-driven protein acting as an outward H^+^ translocating pump which converts light energy to biochemical energy *via* translocation of protons through ATP-proton pumps (Yoshizawa et al., 2012). Both the analyzed psychrotrophic genomes in contrast to the analyzed mesophilic *Flavobacterium* spp. contained a PR-like protein whose activity has been found to enhance growth in some species along with a PR-associated *blh* gene, an essential cofactor of the PR-pump, that encodes 15,15′-β-carotene dioxygenase converting β-carotene to retinal (Peck et al., 2001). PRs were found widely spread among marine psychrophilic bacteria (McCarren and DeLong, 2007) and interestingly, studies on their growth stimulation effects are contradictory in their outcomes (Yoshizawa et al., 2012). Nevertheless, stimulation of growth by a PR-pump was proved in a marine strain belonging to the family *Flavobacteriaceae*, *Dokdonia* sp. MED134 (Kimura et al., 2011), which may suggest that a similar effect can be expected in Antarctic *Flavobacteria* spp., especially as none of these genes were found among the compared mesophilic species.

#### Presence of Prophages and CRISPRs

Both genomes of proposed type strains harbor a significant number of prophages, with half of them predicted as active by genomic prediction tools (Supplementary Tables 6, 7). Interactions between phages and bacterial hosts lead to dynamic co-evolution of the microbial community rather than to the simplest outcome of predation, infection, and lysis of susceptible hosts. This interplay is far more complex and may result in higher adaptation of hosts to environmental factors in a specific niche (Casas and Maloy, 2018). Selective pressure provided by predator-prey relationships can increase overall fitness of a bacterial population by enhancing reproduction rate among infected bacteria (Shapiro et al., 2016), promoting exchange of chromosomal and plasmid DNA (Thierauf et al., 2009) or by lysogenic conversion (Casas and Maloy, 2018). Considering low temperatures in Antarctica negatively influence bacterial growth rate, a possible acceleration of reproduction rates caused by phage infection represents a notable advantage for the hosts. Genetic alterations caused by phages can directly change hosts’ phenotype by acquisition of resistance genes (against antimicrobials or phages/protozoans) or induce metabolic changes (Arnold and Koudelka, 2014; Hurwitz et al., 2015; Gorter et al., 2016; Lee et al., 2017) and thus facilitate the ability of bacterial hosts to adapt and thrive in harsh Antarctic conditions. It is interesting that CRISPR/Cas-systems do not seem to be induced by phage infections in these psychrotrophic *Flavobacterium* strains, since the type II CRISPR/Cas-system was found to be an adaptive immune system in other cold-adapted flavobacteria (Liu et al., 2019). While only the P7388^T^ genome was predicted to contain one CRISPR array without *cas*-associated genes, the remaining analyzed genomes lack *cas*-associated genes as well as CRISPR arrays. This suggests that phage infection of these flavobacteria may be actually advantageous or if they developed defense mechanisms, these are likely associated with the surface receptors alterations, restriction/modification mechanisms or even immunity caused by already present prophages rather than with the Cas-system system (Casas and Maloy, 2018).

#### Resistome

Although routine screening did not find any resistance genes, RGI prediction of the resistome from draft genomes of P4023^T^ and P7388^T^ suggested the presence of multidrug-resistance phenotypes. Ten putative antimicrobial resistance genes (ARGs) were predicted in the P4023^T^ genome likely to provide resistance against 16 different drug classes (Supplementary Table 8). Inactivation is the most abundant mechanism of action among predicted ARGs, followed by target alteration and efflux mechanisms. Although only seven putative ARGs were predicted from the P7388^T^ genome (Supplementary Table 9), they may be active against 20 different drug classes, with inactivation as the main mechanism of action. Furthermore, the predicted resistome did not fully correlate with *in vitro* antibiotic susceptibility testing. Correlation between resistome predictions and *in vitro* testing was found for resistance to aminoglycosides which is presumably encoded by *aadS* gene (MBP4138141.1) in the genome of P7388^T^, a widely spread bacterial adenylyltransferase conferring resistance to aminoglycoside antibiotics (Cox et al., 2015). All Antarctic strains were further found to be resistant or borderline-susceptible to most β-lactams including cephalosporines (Supplementary Table 10). This wide resistance to β-lactam antibiotics may be induced by the gene *JOHN-1* present in P4023^T^ (MBP4142760.1) and P7388^T^ (MBP4137930.1) genomes. This gene provides resistance to a broad spectrum of β-lactam antibiotics in *Flavobacterium johnsoniae* (Naas et al., 2003), however, with a weak effect on susceptibility to carbapenems, which correlates with *in vitro* susceptibility results. Additive effects on resistance to β-lactam antibiotics may result from the presence of putative OXA beta-lactamase *OXA-29* (MBP4140625.1) in P4023^T^, known to hydrolyze penams and cephalosporines but is not effective against carbapenems (Franceschini et al., 2001). Both strains were further predicted to be resistant against tetracyclines, fluoroquinolones, and phenicol; however, *in vitro* tests revealed susceptibility to tetracycline (30 μg), ciprofloxacin (5 μg) and chloramphenicol (30 μg). Disagreement between the phenotypic susceptibility pattern and ARGs computational prediction is a well-known controversy, especially in clinical bacteriology (Hughes and Andersson, 2017). There are several explanations for the phenomenon of phenotype-genotype dissociation which include environmental modulation of gene expression (*in vivo* vs. *in vitro*), testing of single strain culture vs. biofilm formation, presence of specific metabolites and growth factors, presence of specific phages or inadequate time/concentration of tested antibiotics that induce expression of resistance genes. Any of above-mentioned factors or even their combination may be also responsible for discrepancies observed among analyzed Antarctic *Flavobacterium* isolates, in particular considering extreme environment they colonize and presence of phages in their genomes.

Strain P4023^T^ additionally harbors a putative *rosA* gene (MBP4140897.1) encoding an efflux pump/potassium antiporter system. This efflux transporter has been detected in marine *Flavobacterium* spp. (Hao et al., 2018) and within a metagenomic study of Antarctic pristine soils which included *Bacteroidetes* (Van Goethem et al., 2018). The *rosA* gene is related to polymyxin B resistance and represents a part of a two component efflux antiporter system (RosAB) found in *Yersinia enterocolitica* and confers resistance to cationic antimicrobial peptides including polymyxin B (Bengoechea and Skurnik, 2000). So far, no *Flavobacterium* spp. has been detected that could encode a fully functional RosAB system and no data exists to support the effectiveness of this efflux pump without both components. However, all isolated Antarctic strains were resistant or borderline susceptible to polymyxin B suggesting that these strains harbor some unrevealed mechanisms conferring polymyxin B resistance. An environmental study focused on pristine Arctic soils and ancient ARGs revealed that ARGs of soil microbiota are predominantly associated with aminoglycosides resistance and multidrug defense systems including efflux pumps (McCann et al., 2019). Another study focused on Antarctic soils in pristine areas and presence of ancient ARGs clearly showed a high abundance of ARGs which reflects presence of natural antibiotics (Van Goethem et al., 2018). Out of these, efflux pumps were shown as a common trait in a majority of soil microbiota colonizing pristine Antarctic areas which they developed as an adaptive response to diverse chemical stressors (Van Goethem et al., 2018). These findings imply that the polymyxin B resistance of Antarctic *Flavobacterium* spp. may be related to an efflux system, but it can not be concluded at this time that this system is in fact associated with the predicted *rosA* gene.

Further comparison of antibiotic susceptibility profiles of the type strains to the closest related strains showed that they similarly express multidrug resistant phenotypes with susceptibility only to ciprofloxacin, imipenem, co-trimoxazole, and tetracycline. Such results are not surprising as these reference strains are associated with aquaculture (originally isolated from water or fish hosts), which is a well-known environment with resistant bacteria (Cabello et al., 2016). Although the genus *Flavobacterium* comprises numerous species isolated from pristine areas, or various abiotic sources, data on antibiotic susceptibility of environmental isolates are rather scarce. Furthermore, the methods used to determine these antibiotic resistance profiles significantly differs between studies making accurate comparisons challenging for species in this genus. For the moment, the question remains unanswered whether a majority of all *Flavobacterium* spp. express multidrug-resistance phenotypes or if this pattern is simply unique to certain species inhabiting specific niches.

### Morphological, Physiological, and Biochemical Characteristic

Isolates from both groups represented by type strains P4023^T^ and P7388^T^ were aerobic, Gram-negative rods with rounded ends, with average cell size 0.4–0.6 μm × 1.2–2.4 μm and 0.3–0.4 μm × 1.5–3.0 μm, respectively (Supplementary Figures 4, 5). All strains formed yellowish and dark yellow to orange-colored colonies often expressing gliding motility and opalescence on the R2A medium. All strains were catalase positive and oxidase was congruently positive only for strains from the P4023^T^ group. Isolates from both groups can be characterized and distinguished by physiological and biochemical tests listed in Table 2 along with tests useful for their separation from their closest phylogenetic neighbors. Characteristics specific for both groups are given in the description part as well as in a comprehensive formal format in Supplementary Table 11.

**TABLE 2:** Phenotypic characteristics that differentiate *F. flabelliforme* sp. nov and *F. geliluteum* sp. nov. from each other and from their closest phylogenetically related *Flavobacterium* spp. strains.

Tests	*F. flabelliforme* sp. nov.	*F. geliluteum* sp. nov.	1.	2.	3.	4.	5.	6.
**Flexirubin-type of pigments**	–	+	+	+	+	+	+	+
**Growth at:**								
pH 9	+	–	+	w	w	w	+	+
1°C	+	–	+	+	+	+	+	w
5°C	+	v	+	–	–	+	+	+
30°C	+	+	+	+	+	w	+	–
**Growth on marine agar**	+	–	+	–	–	–	–	–
**Growth in presence of:**								
1.0% NaCl	+	–	+	–	+	+	–	+
**Oxidase**	+	–	+	+	–	+	+	–
**Nitrate reduction**	–	–	+	+	–	+	–	+
**Nitrite reduction**	–	–	+	+	–	–	–	–
**Hydrolysis of:**								
Aesculin	–	+	+	+	+	+	+	+
ONPG	–	+	+	+	+	+	+	+
Starch	–	+	+	+	+	+	+	+
Tween 80	–	+	–	–	–	–	–	–
DNA	–	–	+	–	+	+	+	+
Tyrosine	+	+	–	+	+	+	+	–
CMC	–	+	+	–	+	+	–	+
Agar	+	–	–	–	–	–	+	–
**Production of**								
Acid from fructose (aerobically)	–	v	+	+	+	+	+	+
Acid from mannitol (aerobically)	v	–	+	–	–	–	–	+
Acid from xylose (aerobically)	–	+	+	+	+	+	+	+
Arginine dihydrolase	+	–	–	–	+	–	+	–
**Utilization of**								
Arabinose (API 20 NE)	–	+	–	+	+	–	+	+
Mannose (API 20 NE)	–	+	+	+	+	+	+	+
*N*-acetyl-glucosamine (API 20 NE)	–	+	+	–	w	–	+	–
**API ZYM:**								
Valine arylamidase	+	–	+	+	+	+	+	+
Naphtol-AS-BI-phosphohydrolase	–	+	–	–	–	–	–	w
β-galactosidase	–	–	–	–	–	–	+	w
α-glucosidase	–	–	+	–	+	–	+	+
β-glucosidase	–	+	+	+	+	+	+	+
*N*-acetyl-β-glucosaminidase	–	–	–	–	–	–	+	+

*Strains: 1, F. hercynium CCM 9054^T^; 2, F. branchiicola CCM 9061^T^; 3, F. chilense CCM 7940^T^; 4, F. araucananum CCM 7939^T^; 5, F. saccharophilum CCM 8770^T^; 6, F. psychroterrae CCM 8827^T^. +, positive; w, weakly positive; −, negative; v, variable results observed between strains belonging to the same species.*

### Chemotaxonomic Analyses

The analysis of fatty acid methyl esters showed that both groups of Antarctic isolates differ from each other in their major fatty acids (Table 3). The triad comprising strain P4023^T^ revealed five major fatty acids, specifically C_15:1_ ω*6c* (18.1%), Summed Feature 3 (C_16:1_ ω*7c*/C_16:1_ ω*6c*) (9.9%), anteiso-C_15:0_ (9.8%), iso-C_15:0_ (9.3%) and iso-C_16:0_ 3OH (9.3%). The major fatty acid of the group comprising strain P7388^T^ were iso-C_15:0_ (23.4%), Summed Feature 3 (C_16:1_ ω*7c*/C_16:1_ ω*6c*) (9.9%), iso-C_17:0_ 3OH (8.5%) and iso-C_15:0_ 3OH (8.4%). The proposed species differ not only qualitatively and quantitatively in the profiles of major fatty acids, but also quantitatively in minor fatty acids, especially iso-C_14:0_, iso-C_16:1_ H and Summed Feature 9 (C_16:0_ 10-methyl/iso-C_17:1_). The overall fatty acids profile of both groups is in agreement with the genus description (Bernardet and Bowman, 2015) and it is similar to those of the reference strains with mostly quantitative differences. A significant difference is the higher presence of C_15:1_ ω*6c* detected in the P4023^T^ triad that enables its clear separation from the compared strains.

**TABLE 3:**
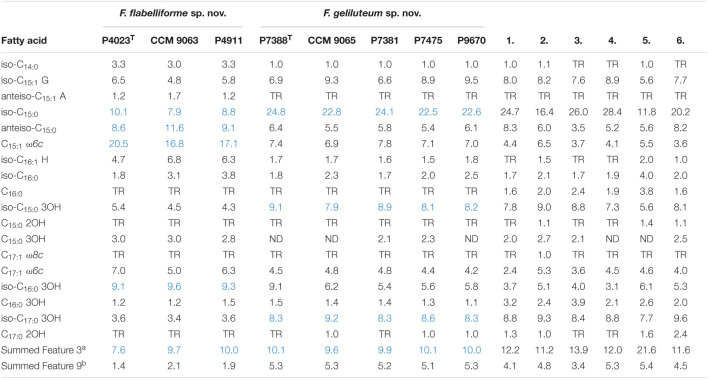
Cellular fatty acids composition (%) of *F. flabelliforme* sp. nov., *F. geliluteum* sp. nov. and their closest phylogenetically related *Flavobacterium* spp.

*^a^C_16:1_ ω7c/C_16:1_ ω6c.*

*^b^C_16:0_ 10-methyl/iso-C_17:1_ ω9c.*

*ND, not detected; TR, trace amounts (<1%); major fatty acids of proposed species are highlighted in blue.*

*Strains: 1, F. hercynium CCM 9054^T^; 2, F. branchiicola CCM 9061^T^; 3, F. chilense CCM 7940^T^; 4, F. araucananum CCM 7939^T^; 5, F. saccharophilum CCM 8770^T^; 6, F. psychroterrae CCM 8827^T^.*

**FIGURE 6:**
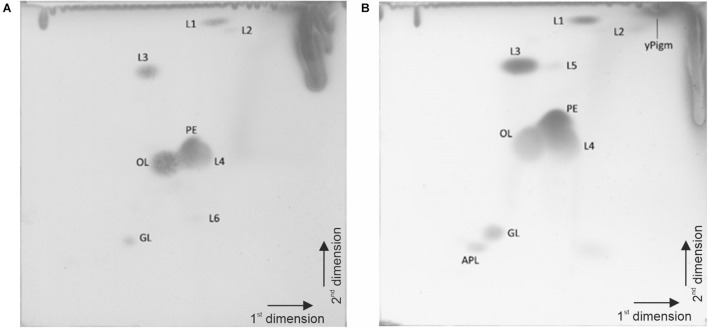
Polar lipid profiles of strains P7388^T^ **(A)** and P4023^T^ **(B)** after two-dimensional thin layer chromatography at detection with 5% ethanolic molybdatophosphoric acid and development at 140°C. PE, phosphatidylethanolamine; OL, ornithine lipid; APL unidentified amino lipid; GL, unidentified glycolipid; L1-5, unidentified lipids lacking a functional group.

The quinone system of strain P4023^T^ was composed of 94.3% menaquinone MK-6, 5.4% MK-5 and 0.2% MK-7, and that of strain P7388^T^ was 90.2% MK-6, 9.6% MK-5 and 0.2% MK-7. The polar lipid profile of strain P7388^T^ (Figure 6A) consisted of the major lipids, phosphatidylethanolamine, an ornithine lipid, two unidentified lipids lacking a functional group (L3, L4), moderate amounts of unidentified lipid L1, minor amounts of lipids L2 and L6 and an unidentified glycolipid (GL). Strain P4023^T^ (Figure 6B) displayed major amounts of phosphatidylethanolamine, an ornithine lipid, and two unidentified lipids (L3, L4) lacking a functional group, moderate amounts of unidentified lipid L1 and glycolipid GL and minor amounts of an unidentified aminophospholipid (APL), and two unidentified lipids (L2, L5). Additionally, a yellow pigment spot was visible (yPigm). The polyamine pattern of strain P4023^T^ consisted of the major polyamine *sym*-homospermidine [27.3 μmol (g dry weight^–1^)] and minor amounts of putrescine [0.7 μmol (g dry weight^–1^)] and spermidine [0.2 μmol (g dry weight^–1^)]. The polyamine pattern of strain P7388^T^ was similar with the major polyamine sym-homospermidine [20.5 μmol (g dry weight^–1^)] and minor amounts of putrescine [0.2 μmol (g dry weight^–1^)] and spermidine [0.3 μmol (g dry weight^–1^)]. Quinone systems, polar lipid profile and polyamine pattern are well in agreement with the genus description (Bernardet et al., 1996; Dong et al., 2013). Both strains differed in their polar lipid profiles from their close relatives *F. hercynium* KACC 14934^T^ and *F. resistens* KACC 14246^T^ (Kim et al., 2012). Specifically, the absence of a lipid labeled PDE (explanation not provided) was noted in the related strains, whereas in the novel strains we detected an aminolipid (OL) with the same chromatographic motility as PDE and corresponded to ornithine lipid 2 (Kawai et al., 1988). The presence of an ornithine lipid or an unidentified aminolipid with the same chromatographic motility as the ornithine lipid in Figure 6 has been reported for numerous *Flavobacterium* species including the type species of the genus *Flavobacterium aquatile* (Kämpfer et al., 2012, 2015, 2020; Lee et al., 2012).

## Conclusion

Eight *Flavobacterium* strains isolated from James Ross Island were analyzed in this study. Using a polyphasic taxonomic approach by combing phenotypic, chemotaxonomic and phylogenomic characteristics, these eight strains forming separated phylogenetic lines were classified as two novel species for which the names *Flavobacterium flabelliforme* sp. nov. and *Flavobacterium geliluteum* sp. nov. are proposed. Comparative genomics analysis showed that the genomes of these Antarctic strains encode numerous genes related to adaptation to cold temperature which is likely a major factor influencing growth in harsh Antarctic conditions. Adaptation mechanisms of Antarctic strains were related to production of pigments, proteorhodopsin, cold-shock proteins, oxidative and osmotic stress, gene expression and membrane fluidity. Although these novel Antarctic species were obtained from an isolated and pristine environment of James Ross Island, they were resistant against a broad spectrum of antimicrobials and predicted to harbor putative resistance genes for a wider spectrum of antimicrobial drugs. This is of particular importance as metagenomic studies have implied that Antarctic microbiota represent a source of ancient antibiotic resistance genes, but so far multidrug resistant isolates were only found in areas with high anthropogenic influence such as King George Island or seawater. The findings in this study thereby makes strains P4023^T^ and P7388^T^ a promising source for future studies on antibiotic resistance genes and resistance mechanisms from environmental microorganisms. Genomic analysis also showed a surprisingly high number of prophage sequences that may influence adaptive response to environmental stresses. In addition, genomes of type strains were predicted to harbor biosynthetic gene clusters with a majority not matching any known BGCs in current databases of biosynthetic genes. These findings suggest that these novel Antarctic psychrophiles are not only interesting taxonomically, but also a promising object of further studies in order to elucidate the effect of phages on their adaptation, the function of their biosynthetic genes, and their antibiotic resistance mechanisms.

## Taxonomy

### Description of *Flavobacterium flabelliforme* sp. nov.

*Flavobacterium flabelliforme* (fla.bel.li.for’me. L. n. *flabellum*, fan, vane; L. suff. *-formis*, -e, -like, in the shape of; N.L. neut. adj. *flabelliforme*, fan-like shaped, referring to fan-like shaped colonies).

Cells are Gram-stain-negative rods with rounded ends, cell size in range 0.4–0.6 μm × 1.2–2.4 μm, cells occurring in irregular clusters, occasionally singly or in pairs. Endospores are not formed. Does not produce a capsule. Negative for presence of flexirubin-type pigments. Does not adhere to agar. Yellowish colonies. Motile with gliding-activity. Catalase and oxidase positive. Growth occurs on R2A, PCA, TSA, marine agar NA, blood agar with 5% sheep blood, BHI, Mueller-Hinton and Endo agar. Does not grow on MacConkey agar. Grows in pH range 6–9 and temperature range 1–30°C with optimum growth at 20°C and pH around 7. Cells grow well in presence of 1% NaCl, and 2% NaCl inhibits growth. Does grow in microaerophilic conditions but growth in anoxic conditions is limited. Does not produce fluorescein on King B medium. No utilization of Simmon’s citrate, malonate and acetamide. Negative for reduction of nitrates and nitrites. Negative for production of urease and indole. Positive for hydrolysis of gelatine, casein, and tyrosine. Does not produce brown diffusible pigment on L-tyrosine agar. Negative for hydrolysis of Tween 80, aesculin, ONPG, starch, DNA, CMC and agar. Does not produce lecithinase. Does not produce H_2_S. Positive for arginine dihydrolase, and negative for ornithine and lysine decarboxylases. Does produce acid from glucose and maltose in aerobic conditions. Negative for production of acid from fructose and xylose in aerobic conditions. Positive for utilization of glucose and maltose by API 20 NE. Negative for utilization of arabinose, mannose, *N*-acetylglucosamine, gluconic acid, capric acid, adipic acid, malic acid, citric acid and phenylacetic acid by API 20 NE. Positive for alkaline phosphatase, leucine arylamidase, valine arylamidase and acid phosphatase by API ZYM. Negative for esterase (C 4), esterase lipase (C 8), lipase (C 14), cystine arylamidase, trypsin, α-chymotrypsin, naphtol-AS-BI-phosphohydrolase, α-galactosidase, β-galactosidase, β-glucuronidase, α-glucosidase, β-glucosidase, *N*-acetyl-β-glucosaminidase, α-mannosidase, and α-fucosidase by API ZYM. Carbon source utilization ability *via* respiration determined by Biolog GEN III MicroPlate test panels is positive for D-maltose, α-D-glucose, D-glucose-6-PO_4_, gelatine, L-arginine, L-aspartic acid, L-glutamic acid, acetoacetic acid and acetic acid. Negative for D-trehalose, D-cellobiose, gentiobiose, sucrose, stachyose, D-raffinose, α-D-lactose, D-melibiose, β-methyl-D-glucoside, D-salicin, *N*-acetyl-β-D-mannosamine, *N*-acetyl neuraminic acid, D-mannose, D-fructose, D-galactose, 3-methyl glucose, D-fucose, L-fucose, L-rhamnose, inosine, D-sorbitol, D-mannitol, D-arabitol, myo-inositol, glycerol, D-fructose-6-PO_4_, D-aspartic acid, D-serine, L-alanine, L-histidine, L-pyroglutamic acid, L-serine, D-galacturonic acid, D-galactonic acid lactone, D-gluconic acid, D-glucuronic acid, glucuronamide, mucic acid, quinic acid, D-saccharic acid, p-hydroxy phenylacetic acid, methyl pyruvate, D-lactic acid methyl ester, L-lactic acid, citric acid, α-keto glutaric acid, D-malic acid, L-malic acid, bromo-succinic acid, Tween 40, γ-amino-butyric acid, α-hydroxy-butyric acid, β-hydroxy-D,L-butyric acid, α-keto butyric acid, propionic acid and formic acid.

The major fatty acids are iso-C_15:1_ ω*6c*, Summed Feature 3 (C_16:1_ ω*7c*/ C_16:1_ ω*6c*), anteiso-C_15:0_, iso-C_15:0_ and iso-C_16:0_ 3OH. Major respiratory quinone is MK-6 and major polyamine is *sym*-homospermidine. Polar lipid profile contains major amounts of phosphatidylethanolamine, an ornithine lipid, and two unidentified lipids (L3, L4) lacking a functional group, moderate amounts of unidentified lipid L1, unidentified glycolipid GL, minor amounts of an unidentified aminophospholipid (APL), and two unidentified lipids (L2, L5). The DNA G + C content of the type strain is 31.2 mol%.

Type strain P4023^T^ (= CCM 9062^T^ = LMG 31963^T^) was isolated in 2011 from the organic material of an abandoned bird nest located at the Lachman Cape (GPS: −63.778333 S, −57.781666 W). All characteristics listed in the species description are shared by all strains, except for the following strain-dependent test results: motility, production of acid from mannitol, and variable results observed by Biolog GEN III MicroPlate were utilization of dextrin, D-turanose, *N*-acetyl-D-glucosamine, glycyl-L-proline and pectin. A formal proposal of the species “*Flavobacterium flabelliforme* sp. nov.” is given in Supplementary Table 11.

The GenBank/EMBL/DDBJ accession numbers for the near full length 16S rRNA gene sequences and whole genome sequences for *Flavobacterium flabelliforme* sp. nov. P4023^T^ are MW691162 and JAGFBU000000000, respectively.

### Description of *Flavobacterium geliluteum* sp. nov.

*Flavobacterium geliluteum* (ge.li.lu’te.um. L. neut. n. *gelum*, cold, frost; L. adj. *luteus*, yellow; N.L. neut. n. *geliluteum*, forming yellow colonies in the cold).

Cells are Gram-stain-negative rods with rounded ends, cell size in range 0.3–0.4 μm × 1.5–3.0 μm, occurring singly and in irregular clusters, occasionally in pairs. Endospores are not formed. Does not produce a capsule. Dark yellow to orange pigmented colonies. Flexirubin-type pigment present. Does not adhere to agar. Motile with gliding-activity. Catalase positive. Oxidase negative. Growth occurs on R2A, PCA, TSA, NA, blood agar with 5% sheep blood, BHI and Mueller-Hinton agar. Does not grow on marine and MacConkey agar. Grows in pH range 6–8 and temperature range 15–30°C with optimum growth at 20°C and pH around 7. Cells grow in presence of 0.5% NaCl, and 1% NaCl inhibits growth. Does grow in microaerophilic conditions but growth in anoxic conditions is limited. Does not produce fluorescein on King B medium. No utilization of Simmon’s citrate, malonate and acetamide. Negative for reduction of nitrates and nitrites. Negative for production of urease and indole. Positive for hydrolysis of gelatine, aesculin, ONPG, starch, casein, tyrosine and CMC. Does not produce brown diffusible pigment on L-tyrosine agar. Negative for hydrolysis of DNA and agar. Does not produce lecithinase. Does not produce H_2_S. Positive for arginine dihydrolase and negative for ornithine and lysine decarboxylases. Does produce acid from glucose, maltose and xylose in aerobic conditions. Negative for production of acid from mannitol in aerobic conditions. Positive for utilization of glucose, arabinose, mannose, *N*-acetyl-glucosamine, maltose, and hydrolysis of aesculin by API 20 NE. Negative for utilization of gluconic acid, capric acid, adipic acid, malic acid, citric acid and phenylacetic acid by API 20 NE. Positive for alkaline phosphatase, leucine arylamidase, acid phosphatase, naphtol-AS-BI-phosphohydrolase and β-glucosidase by API ZYM. Negative for esterase (C 4), esterase lipase (C 8), lipase (C 14), valine arylamidase, cystine arylamidase, trypsin, α-chymotrypsin, α-galactosidase, β-galactosidase, β-glucuronidase, *N*-acetyl-β-glucosaminidase, α-mannosidase and α-fucosidase by API ZYM. Carbon source utilization ability *via* respiration determined by Biolog GEN III MicroPlate test panels is positive for D-trehalose, D-cellobiose, gentiobiose, *N*-acetyl-D-glucosamine, *N*-acetyl-D-galactosamine, α-D-glucose, D-mannose, D-glucose-6-PO_4_, glycyl-L-proline, L-aspartic acid, L-glutamic acid, D-galacturonic acid, acetoacetic acid and acetic acid. Negative for sucrose, stachyose, D-raffinose, α-D-lactose, D-melibiose, *N*-acetyl-β-D-mannosamine, *N*-acetyl neuraminic acid, 3-methyl glucose, D-fucose, L-fucose, L-rhamnose, inosine, D-sorbitol, D-mannitol, D-arabitol, myo-inositol, glycerol, D-aspartic acid, D-serine, L-alanine, D-gluconic acid, D-glucuronic acid, glucuronamide, D-saccharic acid, quinic acid, p-hydroxy phenylacetic acid, L-lactic acid, α-keto glutaric acid, D-malic acid, L-malic acid, bromo-succinic acid, γ-amino-butyric acid, α-hydroxy-butyric acid, β-hydroxy-D,L-butyric acid, α-keto butyric acid, propionic acid, and formic acid.

The major fatty acids are iso-C_15:0_, Summed Feature 3 (C_16:1_ ω*7c*/C_16:1_ ω*6c*), iso-C_17:0_ 3OH and iso-C_15:0_ 3OH. Major respiratory quinone is menaquinone MK-6 and major polyamine is *sym*-homospermidine. Polar lipid profile contains major lipids phosphatidylethanolamine, an ornithine lipid, two unidentified lipids lacking a functional group (L3, L4), moderate amounts of unidentified lipid L1, minor amounts of lipids L2 and L6 and an unidentified glycolipid (GL). The DNA G + C content of the type strain is 34.5 mol%.

Type strain P7388^T^ (= CCM 9064^T^ = LMG 31962^T^) was isolated from water samples taken in 2016 from a small temporary lake (GPS: −63.795894 S, −57.809928 W). All characteristics listed in the species description are shared by all strains, except for the following strain-dependent test results: growth at 5 and 10°C, growth on Endo agar, hydrolysis of Tween 80, production of acid from fructose, presence of α-glucosidase in API ZYM and variable results observed by Biolog GEN III MicroPlate were utilization of dextrin, D-maltose, pectin, β-methyl-D-glucoside, D-salicin, D-fructose, D-galactose, D-fructose-6-PO_4_, gelatine, L-arginine, D-galactonic acid lactone, mucic acid, methyl pyruvate, D-lactic acid methyl ester, citric acid and Tween 40. A formal proposal of the species “*Flavobacterium geliluteum* sp. nov.” is given in Supplementary Table 11.

The GenBank/EMBL/DDBJ accession numbers for the near full length 16S rRNA gene sequences and whole genome sequences for *Flavobacterium geliluteum* sp. nov. P7388^T^ are MW691150 and JAGFBV000000000, respectively.

## Data Availability Statement

The datasets presented in this study can be found in online repositories. The names of the repository/repositories and accession number(s) can be found below: https://www.ncbi.nlm.nih.gov/genbank/, MW691162; https://www.ncbi.nlm.nih.gov/genbank/, JAGFBU000000000; https://www.ncbi.nlm.nih.gov/genbank/, MW691150; https://www.ncbi.nlm.nih.gov/genbank/, JAGFBV000000000.

## Author Contributions

SK performed phylogenetic, phylogenomic, genomic analysis, analysis of fatty acid-methyl esters, and drafted and finalized the manuscript. H-JB performed chemotaxonomic analyses of polar lipids, menaquinones, and polyamines. MB was responsible for whole-genome sequencing. MS-P was involved in genomic analysis of biosynthetic potential. MN was involved in genome assembly and quality of WGS data. DK performed electron microscopy. ES and IS performed morphological, physiological, and biochemical characterization including antibiotic susceptibility. SK drafted the manuscript with inputs of H-JB, MB, MS-P, and IS. All authors edited the draft manuscript and agreed to the final manuscript version for submission.

## Conflict of Interest

The authors declare that the research was conducted in the absence of any commercial or financial relationships that could be construed as a potential conflict of interest.

## Publisher’s Note

All claims expressed in this article are solely those of the authors and do not necessarily represent those of their affiliated organizations, or those of the publisher, the editors and the reviewers. Any product that may be evaluated in this article, or claim that may be made by its manufacturer, is not guaranteed or endorsed by the publisher.

## Footnotes

1
https://polar.sci.muni.cz/en/antarctica

2
http://www.sci.muni.cz/ccm

3
https://eurofinsgenomics.eu/en

4
https://www.ncbi.nlm.nih.gov/

5
http://enve-omics.ce.gatech.edu/ani/

6
http://enve-omics.ce.gatech.edu/aai/

7
https://github.com/tseemann/abricate

8
https://github.com/arpcard/rgi
