# Supplementary material for: Flavobacterium flabelliforme sp. nov. and Flavobacterium geliluteum sp. nov., Two Multidrug-Resistant Psychrotrophic Species Isolated From Antarctica
Source: Front Microbiol. 2021 Oct 22;12:729977. doi: 10.3389/fmicb.2021.729977 (PMC8570120; doi:10.3389/fmicb.2021.729977)
Supplement: Supplementary file 1 [file Data_Sheet_1.pdf]

Supplementary Material

Flavobacterium flabelliforme sp. nov. and *Flavobacterium geliluteum* sp. nov., two multidrug-resistant psychrophilic species isolated from Antarctica

Stanislava Králová[1*], Hans-Jürgen Busse[2], Matěj Bezdíček[3,4], Megan Sandoval-Powers[5], Markéta Nykrýnová[6], Eva Staňková[1], Daniel Krsek[7], Ivo Sedláček[1]

[1] Department of Experimental Biology, Czech Collection of Microorganisms, Faculty of Science, Masaryk University, Brno, Czech Republic

[2] Institut für Mikrobiologie, Veterinärmedizinische Universität Wien, Vienna, Austria

[3] Department of Internal Medicine – Hematology and Oncology, University Hospital Brno, Brno, Czech Republic

[4] Department of Internal Medicine – Hematology and Oncology, Masaryk University, Brno, Czech Republic

[5] Department of Biological Sciences, Auburn University, Auburn, AL, USA

[6] Department of Biomedical Engineering, Faculty of Electrical Engineering and Communication, Brno University of Technology, Brno, Czech Republic

[7] NRL for Diagnostic Electron Microscopy of Infectious Agents, National Institute of Public Health, Prague, Czech Republic

*** Correspondence:**

Stanislava Králová. Phone: +420 606366534. E-mail: kralova.s@sci.muni.cz.

Table S1. List of strains isolated from James Ross Island, Antarctica.

Figure S1. Phylogenetic tree inferred using UBCGs (concatenated alignment of 92 core genes) showing the position of *F. flabelliforme* sp. nov. P4023^T and *F. geliluteum* sp. nov. P7388^T and type strains of the most closely related species of the genus *Flavobacterium*.

Table S2. General features of P4023^T and P7388^T genomes.

Table S3. Clusters of orthologous groups of strains P4023^T and P7388^T.

Figure S2. Superior gliding motility expressed by strain P4023^T on R2A agar after 96 hrs at 15°C.

Figure S3. Lower degree of gliding motility expressed by strain P7388^T on R2A agar after 96 hrs at 15°C.

Table S4. Genes related to gliding activity and T9SS system encoded by P4023^T and P7388^T.

Table S5. Presence of cold-adaptation associated genes in genomes of described psychrophilic species and phylogenetically related mesophilic strains.

Table S6. Putative prophages predicted by PHASTER and Prophage Hunter in the P4023^T genome.

Table S7. Putative prophages predicted by PHASTER and Prophage Hunter in the P7388^T genome.

Table S8. Putative antibiotic resistance genes predicted in the P4023^T genome.

Table S9. Putative antibiotic resistance genes predicted in the P7388^T genome.

Table S10*.* *In vitro* antibiotic susceptibility pattern of Antarctic *Flavobacterium* isolates.

Figure S4. Cellular morphology of strain *Flavobacterium flabelliforme* sp. nov. P4023^T.

Figure S5. Cellular morphology of strain *Flavobacterium geliluteum* sp. nov. P7388^T.

Table S11. Formal descriptions of *Flavobacterium flabelliforme* sp. nov. and *Flavobacterium geliluteum* sp. nov.

Table S1. List of strains isolated from James Ross Island, Antarctica.

Strain	Sample ID	Source	Year of isolation	GPS
P4023^T = CCM 9062^T	64M/5	Material from an abandoned bird nest at the Lachman Cape	2011	-63.778333 S -57.781666 W
CCM 9063	II/2/H	Ornithogenic soil from the Lachman Cape	2010	-63.779197 S -57.781873 W
P4911	V23/4	East side of the Small Lachman Lake	2013	-63.800545 S -57.808623 W
P7388^T = CCM 9064^T	V28/3	Small temporary lake	2016	-63.795894 S -57.809928 W
CCM 9065	V7/2	East side of the Big Lachman Lake	2013	-63.795258 S -57.806070 W
P7381	V27/3	Temporary water pool	2016	-63.798660 S -57.810577 W
P7475	V46/3	Water from mossy swamps near the Dirty Stream	2016	-63.800356 S -57.875403 W
P9670	V50/3	Melted water from the Alpha Glacier	2019	-64.041020 S -57.509406 W

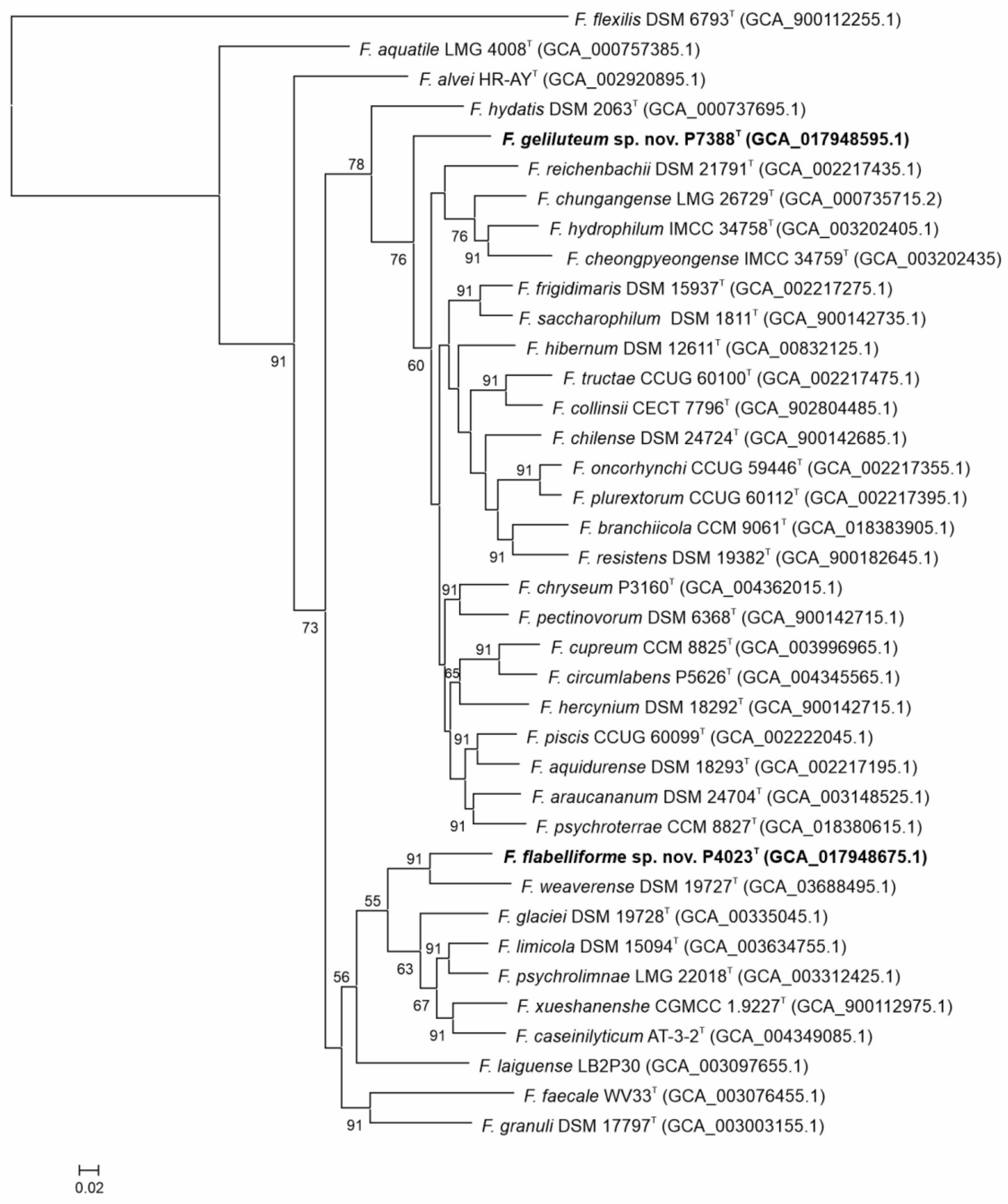

Figure S1. Phylogenetic tree inferred using UBCGs (concatenated alignment of 92 core genes) showing the position of *F. flabelliforme* sp. nov. P4023^{T} and *F. geliluteum* sp. nov. P7388^{T} and type strains of the most closely related species of the genus *Flavobacterium*. *Flexibacter flexilis* DSM 6793^{T} (GCA_900112255.1) was used to root the tree. The number of single gene trees supporting a branch in a UBCG tree is calculated and designated the Gene Support Index (GSI). The GSIs are given at branching points. Bar, 0.02 substitutions per position.

Table S2. General features of P4023^T and P7388^T genomes.

	F. flabelliforme **sp. nov. P4023^T**	***F. geliluteum*** **sp. nov. P7388^T**
GenBank Accession Number	JAGFBU000000000	JAGFBV000000000
Sequencing technology	Illumina MiSeq	Illumina MiSeq
Assembly method	SPAdes v. 3.11.1	SPAdes v. 3.11.1
Genome size (bp)	3,631,245	4,380,917
Mean Coverage (×)	102	88
N50	542,938	115,517
N75	376,317	79,645
L50	2	12
L75	4	23
Largest contig (bp)	1,422,010	373,364
No. of contigs > 1000 bp	22	71
No. of contigs > 10000 bp	11	50
GC content (%)	31.95	34.47
Coding sequences	3,090	3,728
No. of assigned COGs classes	20	20
No. of RNAs	58	52
No. of rRNAs	7	3
No. of tRNAs	48	46
No. of prophages	8	20
No. of plasmids	0	0
CRISPRs	0	1

Table S3. Clusters of orthologous groups of strains P4023^T and P7388^T.

		P4023^T		P7388^T	
**COG class**	**Description**	**Gene count**	**Percentage**	**Gene count**	**Percentage**
	**Information storage and processing**				
A	RNA processing and modification	2	0.07	1	0.03
J	Translation, ribosomal structure and biogenesis	163	5.27	169	4.52
K	Transcription	164	5.30	200	5.35
L	Replication, recombination and repair	159	5.12	190	5.08
	**Metabolism**				
C	Energy production and conversion	145	4.69	148	3.96
E	Amino acid transport and metabolism	214	6.91	239	6.39
F	Nucleotide transport and metabolism	71	2.29	71	1.90
G	Carbohydrate transport and metabolism	94	3.04	188	5.03
H	Coenzyme transport and metabolism	124	4.01	122	3.26
I	Lipid transport and metabolism	101	3.26	134	3.58
P	Inorganic ion transport and metabolism	132	4.26	140	3.75
Q	Secondary metabolites biosynthesis, transport, and catabolism	29	0.94	45	1.20
	**Cellular processes and signalling**				
D	Cell cycle control, cell division, chromosome partitioning	29	0.94	35	0.94
M	Cell wall/membrane/envelope biogenesis	250	8.08	271	7.25
N	Cell motility	13	0.42	22	0.59
O	Post-translational modification, protein turnover, and chaperones	108	3.52	109	2.92
S	Function unknown	667	21.55	796	21.29
T	Signal transduction mechanisms	88	2.84	116	3.10
U	Intracellular trafficking, secretion, and vesicular transport	34	1.10	32	0.86
V	Defence mechanisms	55	1.78	52	1.39
	**Unknown category**				
	COG unknown	359	11.60	485	12.97

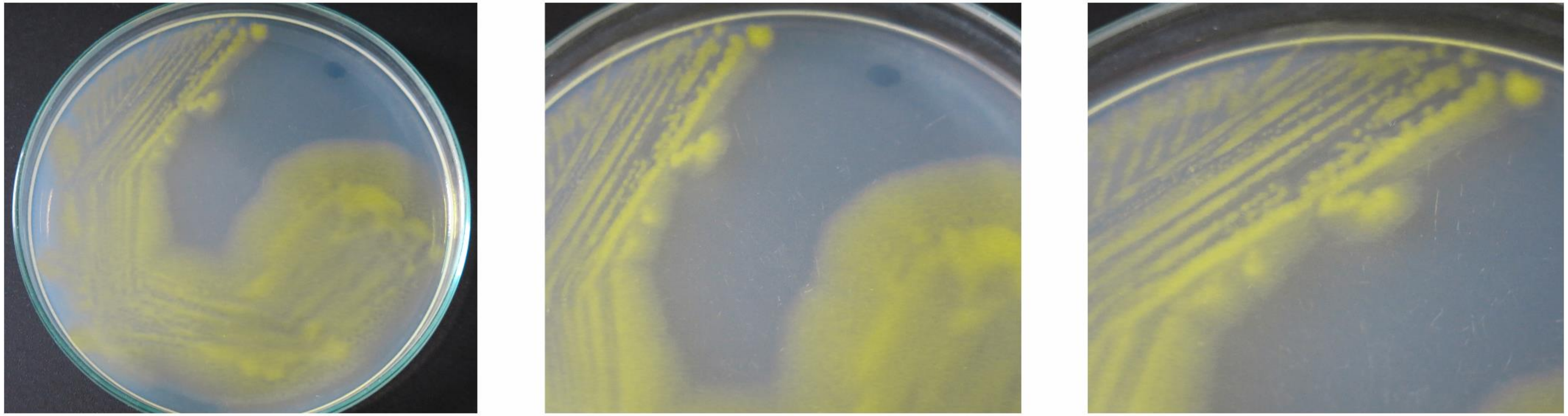

Figure S2. Superior gliding motility expressed by strain P4023^{T} on R2A agar after 96 hrs at 15°C.

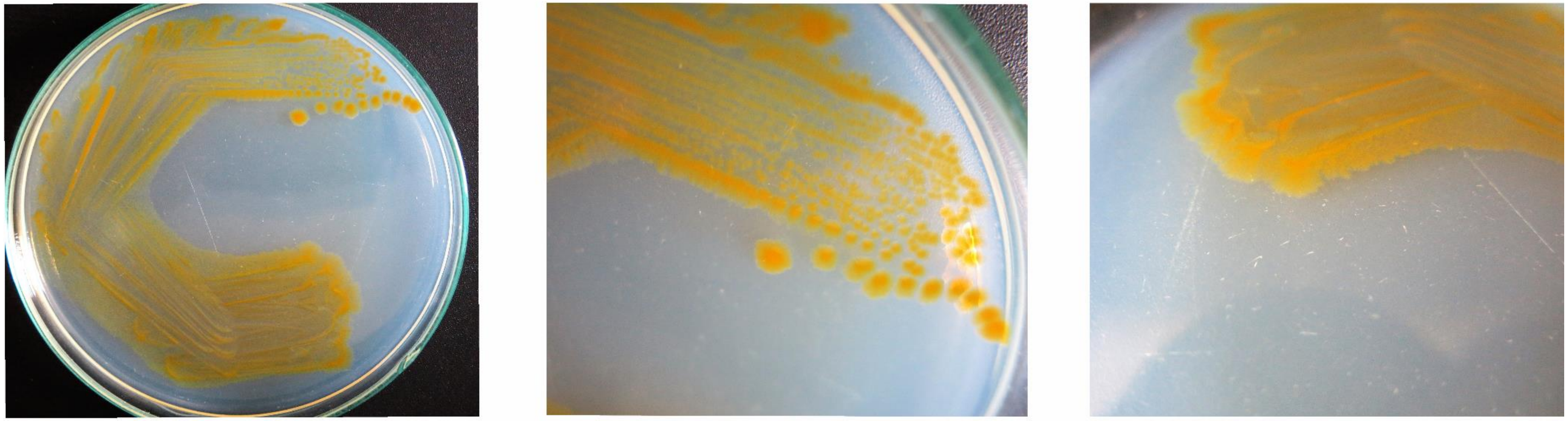

Figure S3. Lower degree of gliding motility expressed by strain P7388^{T} on R2A agar after 96 hrs at 15°C.

Table S4. Genes related to gliding activity and T9SS system encoded by P4023^T and P7388^T.

Gene	Description	Protein Accession Number	
		P4023^T	P7388^T
**Gliding proteins**			
gldA	Gliding motility-associated ABC transporter ATP-binding subunit GldA	MBP4142824.1	MBP4139447.1
gldB	Gliding motility lipoprotein, GldB	MBP4140661.1	MBP4136668.1
gldC	Gliding motility protein, GldC	MBP4140660.1	MBP4136669.1
gldD	Gliding motility protein, GldD	MBP4140636.1	MBP4139451.1
gldE	Gliding motility protein, GldE	MBP4140635.1	MBP4139452.1
gldF	Gliding motility-associated ABC transporter permease protein GldF	MBP4142236.1	MBP4139935.1
gldG	Gliding motility-associated transport system, auxiliary component, GldG	MBP4142235.1	MBP4139934.1
gldH	Gliding motility protein, GldH	MBP4141090.1	MBP4138934.1
gldI	Gliding motility-associated peptidyl-prolyl cis-trans isomerase, GldI	MBP4141642.1	MBP4138454.1
gldJ	Gliding motility protein, GldJ	MBP4141409.1	MBP4136466.1
**Proteins associated with T9SS secretion system**			
gldK	Gliding motility protein, GldK (periplasmic channel component)	MBP4140605.1	MBP4136724.1
gldL	Gliding motility protein, GldL (energizing function)	MBP4140604.1	MBP4136725.1
gldM	Gliding motility protein, GldM (energizing function)	MBP4140603.1	MBP4136726.1
gldN	Gliding motility protein, GldN	MBP4140602.1	MBP4136727.1
sprA	Gliding motility protein, SprA (outer membrane pore)	MBP4140764.1	MBP4136544.1
sprB	Gliding motility protein, SprB	MBP4141916.1	MBP4137444.1
sprC	Gliding motility protein, SprC	-	MBP4138988.1
sprD	Gliding motility protein, SprD	MBP4141134.1	-
sprE	Gliding motility protein, SprE	MBP4141212.1	MBP4137288.1
sprF	Gliding motility protein, SprF	MBP4140743.1	MBP4138986.1
sprT	Gliding motility protein, SprT	MBP4141421.1	MBP4139303.1
porT	PorT family protein	MBP4140411.1	MBP4139086.1
**Attachment complex**			
porQ	Type IX secretion system protein, porQ	MBP4142778.1	MBP4139265.1
porU	Type IX secretion system protein, porU	MBP4141408.1	MBP4136465.1
porV	Transport protein, porV	MBP4141407.1	MBP4136464.1
porX	Two-component system response regulatory protein containing PglZ domain, PorX	MBP4142735.1	MBP4139764.1
**Others**			
remB	Gliding motility protein, RemB	MBP4140760.1	MBP4136548.1

Table S5. Presence of cold-adaptation associated genes in genomes of described psychrophilic species and phylogenetically related mesophilic strains. Strains: 1, P4023^{T}; 2, P7388^{T}; 3, *F. hercynium* DSM 18292^{T}; 4, *F. saccharophilum* DSM 1811^{T}; 5, *F. pectinovorum* DSM 6368^{T}.

Gene categories	Product	COG	1.	2.	3.	4.	5.
**Cold-shock-response**							
cspB	Cold-shock protein	-	+	+	+	+	+
cspC	Cold-shock protein	-	-	+	-	+	+
deaD	Putative cold-shock DEAD-box helicase A	COG0513	+	+	+	+	+
dnaA	Chromosomal replication initiation ATPase DnaA	COG0593	+	+	+	+	+
gyrA	DNA gyrase/topoisomerase IV, subunit A	COG0188	+	+	+	+	+
gyrB	DNA gyrase/topoisomerase IV, subunit B	COG0187	+	+	+	+	+
rpoE	DNA-directed RNA polymerase specialized sigma subunit, sigma24 family	COG1595	++	++	++	++	++
rpoD	DNA-directed RNA polymerase, sigma subunit (sigma70/sigma32)	COG0568	+	+	+	+	+
hupB	Bacterial nucleoid DNA-binding protein HU-beta	COG0776	+	+	+	+	+
nusA	Transcription antitermination factor NusA, contains S1 and KH domains	COG0195	+	+	+	+	+
infA	Translation initiation factor IF-1	COG0361	+	+	+	+	+
infB	Translation initiation factor IF-2, a GTPase	COG0532	+	+	+	+	+
infC	Translation initiation factor IF-3	COG0290	+	+	+	+	+
rbfA	Ribosome-binding factor A	COG0858	+	+	+	+	+
pnp	Polyribonucleotide nucleotidyltransferase (polynucleotide phosphorylase)	COG1185	+	+	+	+	+
rnr	3´-5´ exoribonuclease R	COG0557	+	+	+	+	+
recA	Recombination protein RecA	COG0468	+	+	+	+	+
rplI	Large subunit ribosomal protein L9	COG0359	+	+	+	+	+
rplQ	Large subunit ribosomal protein L17	COG0203	+	+	+	+	+
rho	Transcription termination factor Rho	COG1158	+	+	+	+	+
**Chaperones**							
dnaJ	Molecular chaperone DnaJ (HSP40)	COG0484	+	+	+	+	+
dnaK	Molecular chaperone DnaK (HSP70)	COG0443	+	+	+	+	+
htpG	Molecular chaperone HtpG (HSP90A)	COG0326	+	+	+	+	+
groS	Co-chaperonin GroES (HSP10)	COG0234	+	+	+	+	+
tig	FKBP-type peptidyl-prolyl cis-trans isomerase (trigger factor)	COG0544	+	+	+	+	+

	**Others**						
dus	tRNA-dihydrouridine synthase	-	++	+	+	+	+
	**Oxidative-stress response**						
bcp	Thioredoxin-dependent peroxiredoxin	COG1225	+	+	+	+	+
bsaA	Glutathione peroxidase	COG0386	+	+	+	+	+
ctaC	Cytochrome C oxidase, subunit II	COG1622	+	+	+	+	+
ctaE	Cytochrome C oxidase, subunit III	COG1845	+	+	+	+	+
katA	Catalase	-	+	-	-	+	+
katG	Catalase	COG0376	+	+	+	+	+
katE	Catalase (peroxidase I)	COG0753	+	+	+	+	+
sodA	Superoxide dismutase	COG0605	++++	+	+	+	+
sodC	Cu/Zn superoxide dismutase	COG2032	-	+	+	+	+
tlpA	Alkyl hydroperoxide reductase	-	+	+	+	+	+
trxA	Thioredoxin	COG0526	+++	+	+	+	+
trxA_1	Thioredoxin reductase	-	-	+	-	+	+
trxB	Thioredoxin reductase	COG0492	+	+	+	+	+
trxB2	Thioredoxin reductase	-	-	+	+	+	+
osmC	Organic hydroperoxide reductase OsmC (peroxiredoxin)	COG1764	+++	+++	+++	+++	+++
osmC-like	Putative peroxiredoxin	-	+++	++++	+++	+++	++++
	**Membrane-associated alterations**						
**Proteorhodopsin**							
PR-like	Proteorhodopsine-like protein	-	+	+	-	-	-
blh	β-carotene 15,15’-dioxygenase	-	+	+	-	-	-
**Carotenoids**							
crtB	Phytoene synthase	-	+	+	+	+	+
crtI	Phytoene desaturase/dehydrogenase	COG1233	+	+	+	+	+
crtZ	Beta-carotene 3-hydroxylase	-	+	-	-	-	-
crtY	Lycopene beta-cyclase	-	+	+	+	+	+
idi	Isopentenyl-diphosphate Delta-isomerase	COG1443	+	+	+	+	+
**Other membrane alterations**							
desA1	Acyl-[acyl-carrier-protein] desaturase	-	+	+	+	+	+
wza	Polysaccharide biosynthesis/export protein	COG1596	+	+	+	+	+

		**Exopolysaccharides related genes**						
	ptB	Lipopolysaccharide export system ATP-binding protein	COG1137	+	+	+	+	+
	lptC	Lipopolysaccharide export system protein LptC	COG3117	+	+	+	+	+
	lptE	LPS-assembly lipoprotein lptE	-	+	+	+	+	+
	lptG	Lipopolysaccharide export system permease protein	COG0795	+	+	+	+	+
		**Osmoprotection**						
**Proline metabolism**								
	proA	Gamma-glutamyl phosphate reductase	COG0014	-	+	-	+	+
	proB	Glutamate 5-kinase	COG0263	-	+	-	+	+
	proC	Pyrroline-5-carboxylate reductase	COG0345	-	+	-	+	+
	putP	NA^{+}/proline symporter	COG0591	-	-	-	-	-
**Glycogen metabolism**								
	glgA	Glycogen synthase	COG0297	+	+	+	+	++
	glgB	1,4-alpha-glucan branching enzyme	COG0296	-	+	+	+	+
	glgC	ADP-glucose pyrophosphorylase	COG0448	-	+	+	+	+

Table S6. Putative prophages predicted by PHASTER and Prophage Hunter in the genome of strain P4023^{T}.

		**PHASTER**			
**ID**	**Scaffold**	**Start**	**End**	**Category**	**No. of genes**
P1	14	125	5511	questionable*	6
		**Prohage Hunter**			
**ID**	**Scaffold**	**Start**	**End**	**Category**	**No. of genes**
P2	1	242895	264326	ambiguous¥	25
P3	1	256876	267957	active	11
P4	1	486119	497492	ambiguous	9
P5	2	487162	498830	ambiguous	10
P6	3	1254	14411	active	13
P7	3	363566	383387	active	21
P8	10	2596	12970	active	11

*PHASTER categories: intact/questionable/incomplete, ¥Prophage Hunter categories: active/ambiguous/inactive

Table S7. Putative prophages predicted by PHASTER and Prophage Hunter in the genome of strain P7388^T.

PHASTER					
**ID**	**Scaffold**	**Start**	**End**	**Category**	**No. of genes**
PP1	5	44370	52892	incomplete*	7
PP2	8	68943	78078	incomplete	10
PP3	8	86623	98865	incomplete	11
PP4	10	120355	125594	incomplete	6
PP5	13	16701	26840	incomplete	9
PP6	45	2766	12545	incomplete	12
**Prohage Hunter**					
**ID**	**Scaffold**	**Start**	**End**	**Category**	**No. of genes**
PP7	1	241057	632175	active¥	15
PP8	5	165715	190078	active	33
PP9	6	2586	17990	ambiguous	10
PP10	8	53531	89479	ambiguous	37
PP11	14	17313	35437	ambiguous	16
PP12	16	1250	24962	ambiguous	22
PP13	23	21713	43755	ambiguous	15
PP14	29	24326	36750	ambiguous	10
PP15	31	2207	19815	active	22
PP16	31	5624	24314	active	31
PP17	32	32116	47672	ambiguous	15
PP18	33	1117	13282	ambiguous	10
PP19	43	498	17257	active	31
PP20	45	599	16249	active	21

*PHASTER categories: intact/questionable/incomplete, ¥Prophage Hunter categories: active/ambiguous/inactive

Table S8. Putative antibiotic resistance genes predicted in the P4023^T genome.

Protein Accession Number	Best hit to Antibiotic Resistance Ontology (ARO)	ARO	Sequence similarity (%)	AMR gene family	Drug class	Resistance mechanism
MBP4140486.1	*Escherichia coli* EF-Tu mutants conferring resistence to Pulvomycin	3003369	69.87	elfamycin resistant EF-Tu	elfamycin antibiotic	antibiotic target alteration
MBP4140625.1	OXA-29	3001424	64.29	OXA beta-lactamase	cephalosporin; penam	antibiotic inactivation
MBP4140823.1	*abeS*	3000768	63.21	small multidrug resistance (SMR) antibiotic efflux pump	macrolide antibiotic; aminocoumarine antibiotic	antibiotic efflux
MBP4140413.1	*iri*	3002884	61.11	rifampin monooxygenase	rifamycin antibiotic	antibiotic inactivation
MBP4142858.1	*Mycobacterium tuberculosis thyA* with mutation conferring resistance to para-aminosalicylic acid	3004153	58.48	aminosalicylate resistant thymidylate synthase	para-aminosalicylic acid	antibiotic target alteration
MBP4142760.1	*JOHN-1*	3000840	54.7	JOHN beta-lactamase	carbapenem; cephalosporin; penam	antibiotic inactivation
MBP4142391.1	*AAC(3)-IIb*	3002534	52.94	AAC(3)	aminoglycoside antibiotic	antibiotic inactivation
MBP4140289.1	*vatB*	3002841	52.63	streptogramin vat acetyltransferase	streptogramin antibiotic	antibiotic inactivation
MBP4140897.1	*rosA*	3003048	51.35	major facilitator superfamily (MFS) antibiotic efflux pump	peptide antibiotic	antibiotic efflux
MBP4143105.1	*vgaC*	3002831	50.0	ABC-F ATP-binding cassette ribosomal protection protein	macrolide antibiotic; lincosamide antibiotic; streptogramin antibiotic; tetracycline antibiotic; oxazolidine antibiotic; phenicol antibiotic; pleuromutilin antibiotic	antibiotic target protection

Table S9. Putative antibiotic resistance genes predicted in the P7388^{T} genome.

Protein Accession Number	Best hit to Antibiotic Resistance Ontology (ARO)	ARO	Sequence similarity (%)	AMR gene family	Drug class	Resistance mechanism
MBP4137930.1	*JOHN-1*	3000840	77.82	JOHN beta-lactamase	carbapenem; cephalosporin; penam	antibiotic inactivation
MBP4138898.1	*Escherichia coli* EF-Tu mutants conferring resistence to Pulvomycin	3003369	69.62	elfamycin resistant EF-Tu	elfamycin antibiotic	antibiotic target alteration
MBP4138431.1	*Mycobacterium tuberculosis katG* with mutation conferring resistance to isoniazid	3003392	62.9	isoniazid resistant katG	isoniazid	antibiotic target alteration
MBP4136558.1	*oprM*	3000379	62.5	resistance-nodulation-cell division (RND) antibiotic efflux pump	macrolide antibiotic; fluoroquinolone antibiotic; monobactam; aminoglycoside antibiotic; carbapenem; cephalosporin; cephamycin; penam; tetracycline antibiotic; peptide antibiotic; acridine dye; aminocoumarine antibiotic; diaminopyrimidine antibiotic; sulphonamide antibiotic; Phenicol antibiotic; penem	antibiotic efflux
MBP4136569.1	*PEDO-2*	3003714	52.17	subclass B3 PEDO beta lactamase	carbapenem	antibiotic inactivation
MBP4137502.1	*vgaC*	3002831	50.0	ABC-F ATP-binding cassette ribosomal protection protein	macrolide antibiotic; lincosamide antibiotic; streptogramin antibiotic; tetracycline antibiotic; oxazolidine antibiotic; phenicol antibiotic; pleuromutilin antibiotic	antibiotic target protection
MBP4138141.1	*aadS*	3004683	50.0	ANT(6)	aminoglycoside antibiotic	antibiotic inactivation

.

Table S10. *In vitro* antibiotic susceptibility pattern of Antarctic *Flavobacterium* isolates and their closest phylogenetic relatives.

Strains: 1, *F. hercynium* CCM 9054^{T}; 2, *F. branchiicola* CCM 9061^{T}; 3, *F. chilense* CCM 7940^{T}; 4, *F. araucananum* CCM 7939^{T}; 5, *F. saccharophilum* CCM 8770^{T}; 6, *F. psychtorerrae* CCM 8827^{T}

**ATB susceptibility**	***F. flabelliforme* sp. nov.**			***F. geliluteum* sp. nov.**					**1.**	**2.**	**3.**	**4.**	**5.**	6.
	P4023^{T}	CCM 9063	P4911	P7388^{T}	CCM 9065	P7381	P7475	P9670						
Ampicillin (10 µg)	S	S	S	R	R	R	R	R	R	R	R	R	R	R
Aztreonam (30 µg)	R	R	R	R	R	R	R	R	R	R	R	R	R	R
Carbenicillin (100 µg)	R	R	I	R	R	R	R	R	R	R	R	R	R	R
Cefixime (5 µg)	R	R	R	R	R	R	R	R	R	R	R	R	R	R
Ceftazidime (10 µg)	R	R	R	R	R	R	R	R	R	R	R	R	R	R
Cephalothin (30 µg)	R	R	I	R	I	I	R	R	R	R	R	R	R	R
Ciprofloxacin (5 µg)	S	S	S	S	S	S	S	S	S	S	S	S	S	S
Gentamicin (10 µg)	I	S	S	R	R	R	R	R	R	R	R	R	R	R
Chloramphenicol (30 µg)	S	S	S	S	S	S	S	S	S	R	R	R	R	S
Imipenem (10 µg)	S	S	S	S	S	S	S	S	S	S	S	S	S	S
Kanamycin (30 µg)	S	S	S	R	R	R	R	R	R	R	R	R	R	R
Co-trimoxazole (25 µg)	S	S	S	S	S	S	S	S	S	S	S	S	S	R
Piperacillin (30 µg)	R	R	I	R	R	R	R	R	R	R	R	R	R	R
Polymyxin B (300 U)	S	R	R	I	I	I	I	R	R	R	R	R	R	S
Streptomycin (10 µg)	S	S	S	R	R	R	R	R	R	R	R	R	R	R
Tetracycline (30 µg)	S	S	S	S	S	S	S	S	S	S	S	S	S	S

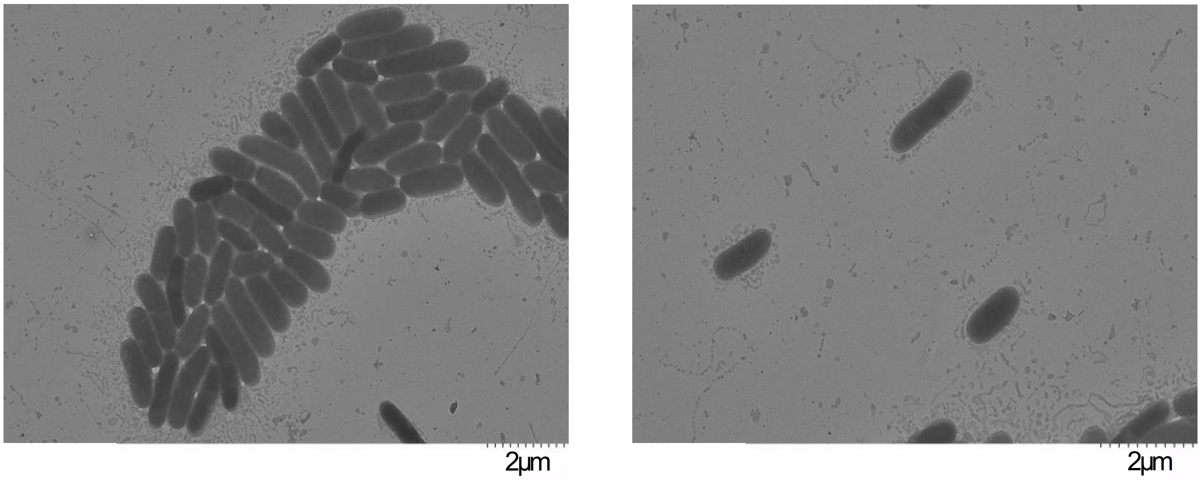

Figure S4. Cellular morphology of strain *Flavobacterium flabelliforme* sp. nov. P4023^T cultivated on R2A agar (Oxoid). Images were obtained using transmission electron microscopy performed with a Morgagni 268D Philips (FEI Company, USA) electron microscope. Negative staining with 2% ammonium molybdate.

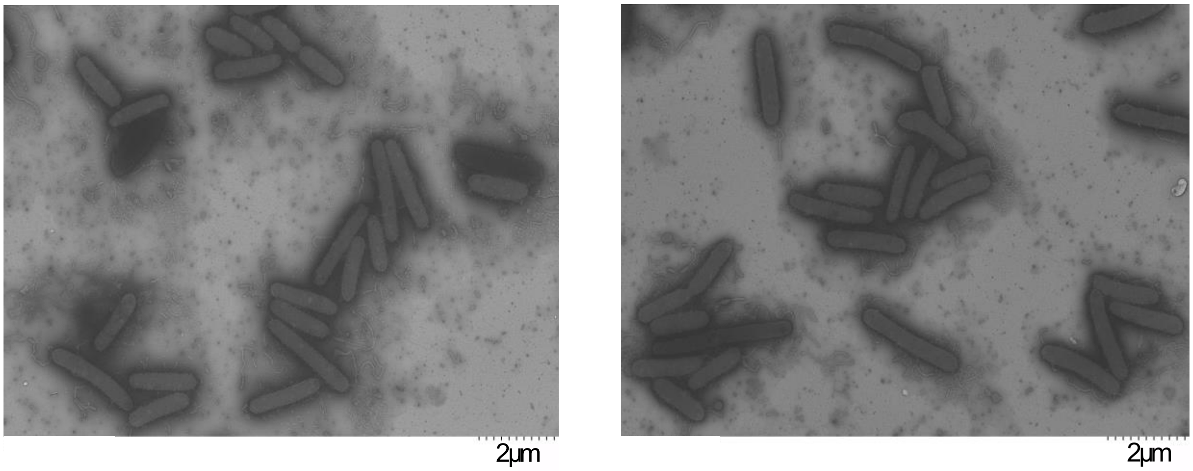

Figure S5. Cellular morphology of strain *Flavobacterium geliluteum* sp. nov. P7388^T cultivated on R2A agar (Oxoid). Images were obtained using transmission electron microscopy performed with a Morgagni 268D Philips (FEI Company, USA) electron microscope. Negative staining with 2% ammonium molybdate.

Table S11: Formal descriptions of *Flavobacterium flabelliforme* sp. nov. and *Flavobacterium geliluteum* sp. nov.

**Genus name**	*Flavobacterium*	*Flavobacterium*
**Species name**	*Flavobacterium flabelliforme*	*Flavobacterium geliluteum*
**Specific epithet**	*flabelliforme*	*geliluteum*
**Species status**	sp. nov.	sp. nov
**Species etymology**	fla.bel.li.for'me. L. n. *flabellum*, fan, vane; L. suff. -*formis*, -e, -like, in the shape of; N.L. neut. adj. *flabelliforme*, fan-like shaped, referring to fan-like shaped colonies	ge.li.lu'te.um, L. neut. n. *gelum*, cold, frost; L. adj. *luteus*, yellow; N.L. neut. n. *geliluteum*, forming yellow colonies in the cold
**Description of the new taxon and diagnostic traits**	**Phenotypic characteristics:**<br>• Gram-negative rods with rounded ends, size 0.4-0.6 x 1.2-2.4 µm<br>• microscopically: irregular clusters, occasionally single cells and pairs<br>• endospores are not produced<br>• does not produce capsules (Congo red adsorption test)<br>• does not adhere to agar<br>• flexirubin-type of pigments not produced<br>• yellowish colonies, often expressing gliding motility<br>• temperature range 1-30 °C, optimum 20°C<br>• pH range 6-9, optimum 7<br>• grows at 0-1 % NaCl, 2% NaCl inhibits growth<br>• limited growth in anoxic conditions<br>• does grow in microaerophilic conditions<br>• catalase and oxidase positive<br>• growth on R2A, PCA, TSA, marine agar, NA, blood agar with 5% sheep blood, BHI, Mueller-Hinton and Endo agar<br>• no growth on MacConkey agar<br>• does not produce fluorescein on King B medium<br>• no utilization of Simmon´s citrate, malonate and acetamide<br>• negative for reduction of nitrates and nitrites<br>• negative for production of urease and indole<br>• positive for hydrolysis of gelatine, casein, and tyrosine<br>• negative for hydrolysis of Tween 80, aesculin, ONPG, starch, DNA, carboxymethylcellulose and agar<br>• does not produce brow diffusible pigment on L-tyrosine agar	**Phenotypic characteristics:**<br>• Gram-negative rods with rounded ends, size 0.3-0.4 x 1.5-3.0 µm<br>• microscopically: irregular clusters, occasionally single cells and pairs<br>• endospores are not produced does not produce capsules (Congo red adsorption test)<br>• does not adhere to agar<br>• flexirubin-type of pigments produced<br>• dark yellow to orange colonies, often expressing gliding motility<br>• temperature range 15-30 °C, optimum 20°C<br>• pH range 6-8, optimum 7<br>• grows at 0-0.5 % NaCl, 1% NaCl inhibits growth<br>• limited growth in anoxic conditions<br>• does grow in microaerophilic conditions<br>• catalase positive and oxidase negative<br>• growth on R2A, PCA, TSA, NA, blood agar with 5% sheep blood, BHI and Mueller-Hinton<br>• no growth on marine and MacConkey agar<br>• does not produce fluorescein on King B medium<br>• no utilization of Simmon´s citrate, malonate and acetamide<br>• negative for reduction of nitrates and nitrites<br>• negative for production of urease and indole<br>• positive for hydrolysis of gelatine, aesculin, ONPG, starch, casein, tyrosine and carboxymethylcellulose<br>• negative for hydrolysis of DNA and agar<br>• does not produce brown diffusible pigment on L-tyrosine agar

- does not produce lecithinase
- does not produce H_2S
- positive for arginine dihydrolase and negative for ornithine and lysine decarboxylases
- does produce acid from glucose and maltose in aerobic conditions
- does not produce acid from fructose and xylose in aerobic conditions
- API 20 NE positive: utilization of glucose and maltose
- API 20 NE negative: utilization of arabinose, mannose, N-acetyl-glucosamine, gluconic acid, capric acid, adipic acid, malic acid, citric acid and phenylacetic acid
- API ZYM positive: alkaline phosphatase, leucine arylamidase, valine arylamidase and acid phosphatase
- API ZYM negative: esterase (C 4), esterase lipase (C 8), lipase (C 14), cystine arylamidase, trypsin, α-chymotrypsin, naphtol-AS-BI-phosphohydrolase, α-galactosidase, β-galactosidase, β-glucuronidase, α-glucosidase, β-glucosidase, N-acetyl-β-glucosaminidase, α-mannosidase and α-fucosidase
- Biolog GEN III MicroPlate positive tests: D-maltose, α-D-glucose, D-glucose-6-PO_4, gelatin, L-arginine, L-aspartic acid, L-glutamic acid, acetoacetic acid and acetic acid
- Biolog GEN III MicroPlate negative tests: D-trehalose, D-cellobiose, gentiobiose, sucrose, stachyose, D-raffinose, α-D-lactose, D-melibiose, β-methyl-D-glucoside, D-salicin, N-acetyl-β-D-mannosamine, N-acetyl neuraminic acid, D-mannose, D-fructose, D-galactose, 3-methyl glucose, D-fucose, L-fucose, L-rhamnose, inosine, D-sorbitol, D-mannitol, D-arabitol, myo-inositol, glycerol, D-fructose-6-PO_4, D-aspartic acid, D-serine, L-alanine, L-histidine, L-pyroglutamic acid, L-serine, D-galacturonic acid, D-galactonic acid lactone, D-gluconic acid, D-glucuronic acid, glucuronamide, mucic acid, quinic acid, D-saccharic acid, p-hydroxy phenylacetic acid, methyl pyruvate, D-lactic acid methyl ester, L-lactic acid, citric acid, α-keto glutaric acid, D-malic acid, L-malic acid, bromo-succinic acid, Tween 40, γ-amino-butyric acid, α-hydroxy-butyric acid, β-hydroxy-D,L-butyric acid, α-keto butyric acid, propionic acid, formic acid

- does not produce lecithinase
- does not produce H_2S
- positive for arginine dihydrolase and negative for ornithine and lysine decarboxylases
- does produce acid from glucose, maltose, and xylose in aerobic conditions
- does not produce acid from mannitol in aerobic conditions
- API 20 NE positive: utilization of glucose, arabinose, mannose, N-acetyl-glucosamine and maltose, hydrolysis of aesculin
- API 20 NE negative: utilization of gluconic acid, capric acid, adipic acid, malic acid, citric acid and phenylacetic acid
- API ZYM positive: alkaline phosphatase, leucine arylamidase, acid phosphatase, naphtol-AS-BI-phosphohydrolase and β-glucosidase
- API ZYM negative: esterase (C 4), esterase lipase (C 8), lipase (C 14), valine arylamidase, cystine arylamidase, trypsin, α-chymotrypsin, α-galactosidase, β-galactosidase, β-glucuronidase, N-acetyl-β-glucosaminidase, α-mannosidase and α-fucosidase
- Biolog GEN III MicroPlate positive tests: D-trehalose, D-cellobiose, gentiobiose, N-acetyl-D-glucosamine, N-acetyl-D-galactosamine, α-D-glucose, D-mannose, D-glucose-6-PO_4, glycyl-L-proline, L-aspartic acid, L-glutamic acid, D-galacturonic acid, acetoacetic acid and acetic acid
- Biolog GEN III MicroPlate negative tests: sucrose, stachyose, D-raffinose, α-D-lactose, D-melibiose, N-acetyl-β-D-mannosamine, N-acetyl neuraminic acid, 3-methyl glucose, D-fucose, L-fucose, L-rhamnose, inosine, D-sorbitol, D-mannitol, D-arabitol, myo-inositol, glycerol, D-aspartic acid, D-serine, L-alanine, D-gluconic acid, D-glucuronic acid, glucuronamide, D-saccharic acid, quinic acid, p-hydroxy phenylacetic acid, L-lactic acid, α-keto glutaric acid, D-malic acid, L-malic acid, bromo-succinic acid, γ-amino-butyric acid, α-hydroxy-butyric acid, β-hydroxy-D,L-butyric acid, α-keto butyric acid, propionic acid, formic acid

	**Chemotaxonomic characteristics:**<br>• major fatty acids: $C_{15:1}$ *ω6c,* Summed Feature 3 ($C_{16:1}$ *ω7c*/ $C_{16:1}$ *ω6c*), anteiso-$C_{15:0}$, iso-$C_{15:0}$ and iso-$C_{16:0}$ 3OH<br>• major respiratory quinone is MK-6<br>• major polyamine is *sym*-homospermidine<br>• major lipids are phosphatidylethanolamine, an ornithine lipid, and two unidentified lipids (L3, L4) lacking a functional group, followed by moderate amounts of unidentified lipid L1, unidentified glycolipid GL, minor amounts of an unidentified aminophospholipid (APL), and two unidentified lipids (L2, L5)	**Chemotaxonomic characteristics:**<br>• major fatty acids iso-$C_{15:0}$, Summed Feature 3 ($C_{16:1}$ *ω7c*/ $C_{16:1}$ *ω6c*), iso-$C_{17:0}$ 3OH and iso-$C_{15:0}$ 3OH<br>• major respiratory quinone is MK-6<br>• major polyamine is *sym*-homospermidine<br>• major lipids are phosphatidylethanolamine, an ornithine lipid, two unidentified lipids lacking a functional group (L3, L4), followed by moderate amounts of unidentified lipid L1, minor amounts of lipids L2 and L6 and an unidentified glycolipid (GL)
**Country of origin**	Antarctica	Antarctica
**Region of origin**	James Ross Island	James Ross Island
**Type strain information:**		
**Date of isolation**	11.02.2011	14.01.2016
**Source of isolation**	organic material of an abandoned bird nest	small temporary lake
**Sampling date**	08.02.2011	12.01.2016
**Latitude**	63° 46′ 42″ S	63° 47′ 45″ S
**Longitude**	57° 46′ 54″ W	57° 48′ 36″ W
**Altitude**	1 630 m	1 630 m
**16S rRNA gene accession nr.**	MW691162	MW691150
**Genome accession number**	JAGFBU000000000	JAGFBV000000000
**Genome status**	incomplete	incomplete
**Genome size**	3,65 Mbp	4,39 Mbp
**GC mol%**	31.2	34.5
**Number of strains in study**	3	5
**Source of isolation of non-type strains**	soil and water sources	various water sources
**Information related to the Nagoya Protocol**	Antarctica falls outside the scope of Nagoya Protocol	Antarctica falls outside the scope of Nagoya Protocol
**Designation of the Type Strain**	P4023^T	P7388^T
**Strain Collection Numbers**	CCM 9062^T = LMG 31963^T	CCM 9064^T = LMG 31962^T
